# Vitiligo as a Failure of Immune Resolution and Tissue Regeneration: From Stress Signals to Targeted Immune Modulation

**DOI:** 10.1007/s12016-026-09165-3

**Published:** 2026-05-09

**Authors:** Ana Vitória Pupo Silvestrini, Jackeline Marino Lucas, Cristina Ribeiro de Barros Cardoso, Maria Vitória Lopes Badra Bentley

**Affiliations:** 1 School of Pharmaceutical Sciences of Ribeirao Preto, University of Sao Paulo, Ribeirao Preto, SP Brazil; 2 Faculty of Medicine of Ribeirao Preto, University of Sao Paulo, Av. do Café, s/n, 14040–903, Ribeirao Preto, SP Brazil

**Keywords:** Vitiligo, Autoimmunity, Targeted therapy, Precision medicine, Regeneration, Immunosuppression

## Abstract

Traditionally, vitiligo is known as a cutaneous autoimmune disease characterized by progressive melanocyte loss. However, this paradigm alone does not fully explain disease chronicity, relapse, or the limited therapeutic responses. Evidence supports a broader conceptual framework in which vitiligo is sustained by defective immune control of dysregulated responses, coupled with impaired regeneration, resulting in persistent inflammation and failure to restore cutaneous homeostasis. Here, we reinterpret vitiligo immunopathogenesis as a disorder of failed restoration of immunological homeostasis, extending beyond classical immune activation to include defects in regulatory mechanisms that normally constrain autoreactive responses. Rather than focusing exclusively on broad immunosuppression, emerging strategies increasingly aim to restore local immune regulation, constrain pathogenic immune memory, and preserve or reconstitute melanocyte regenerative niches. Advances in cutaneous immunology and biotechnology have expanded the therapeutic landscape to include targeted cytokine and kinase inhibition, immune checkpoint modulation, pro-melanogenic and regenerative approaches, cell- and vesicle-based therapies, nucleic acid-based interventions, and advanced skin delivery systems. Accumulating knowledge supports combined and sequential treatment paradigms integrating immune modulation, induction of repigmentation, and long-term maintenance. Nevertheless, translation into routine clinical practice remains limited by cutaneous pharmacokinetic barriers, regulatory and manufacturing constraints, and the lack of long-term safety and efficacy data. Within this evolving landscape, precision medicine offers a framework for patient stratification, therapeutic timing, and rational treatment design. Importantly, many of these approaches remain in early or preclinical stages, highlighting the need for robust translational validation. On the other hand, delivery platforms based on nanotechnology emerge as potential tools capable of bridging diverse therapeutic modalities by enhancing stability, targeting, and cutaneous bioavailability. Finally, this review positions vitiligo as a disease of failed immune resolution and impaired tissue regeneration, highlighting precision immunoengineering and technological innovation as promising pathways toward more targeted, personalized, and durable disease-modifying therapies.

## Introduction

Vitiligo has been documented since the second millennium BC and affects millions of people worldwide. Throughout history, achromic skin patches were frequently grouped with conditions such as malaria and leprosy and interpreted in many cultures as signs of spiritual impurity or divine punishment. These perceptions often led to social stigmatization and the segregation of affected individuals, contributing to the enduring psychosocial burden associated with the disease [1].

**Fig. 1:**
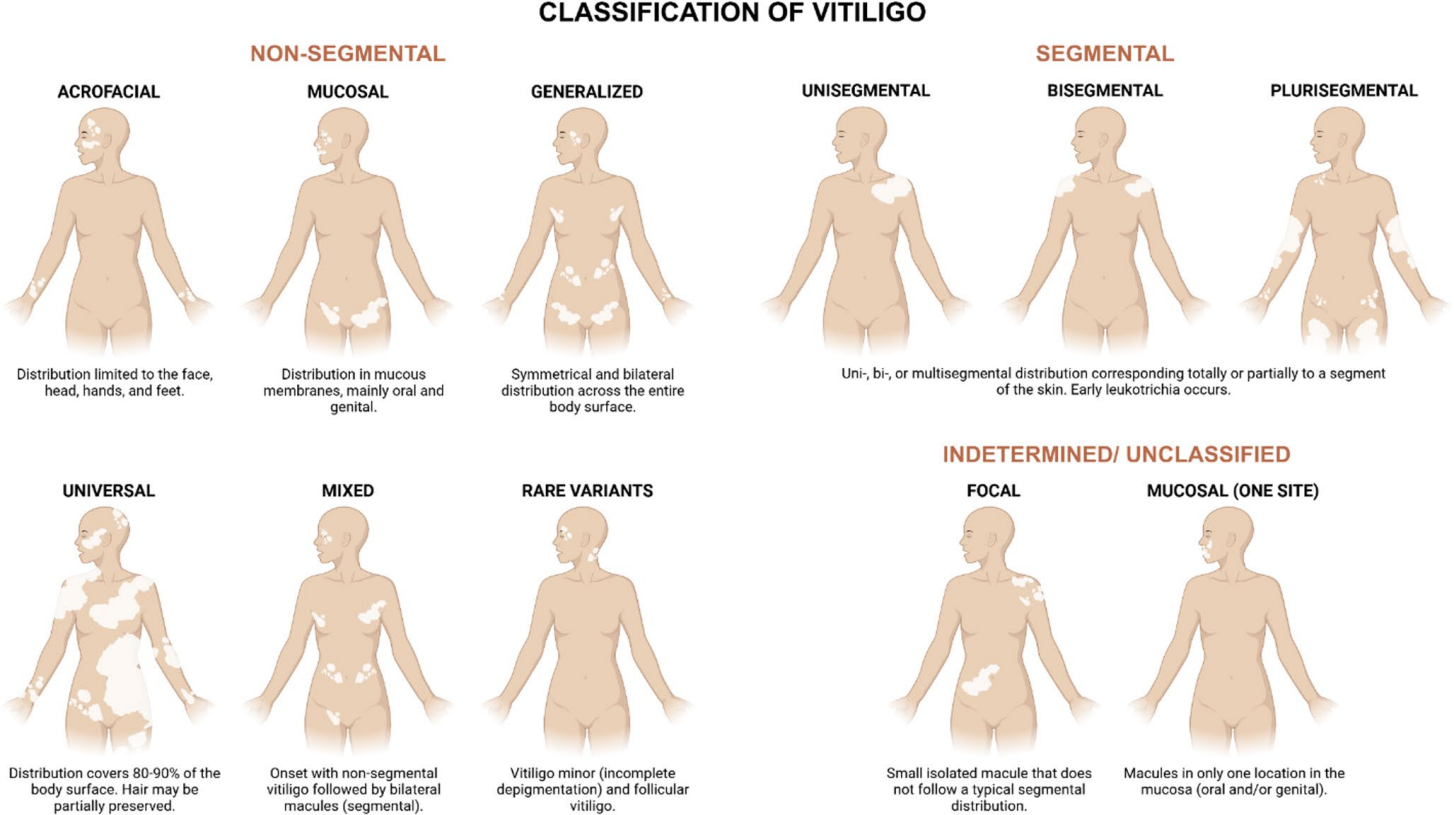
Schematic representation of the main clinical categories of vitiligo, encompassing non-segmental forms (acrofacial, mucosal, generalized, universal, mixed, and uncommon variants), segmental vitiligo (uni-, bi-, or multisegmental), and indeterminate/unclassified presentations (focal and single-site mucosal), with emphasis on their typical distribution patterns and defining clinical characteristics. Created in BioRender

From a biomedical perspective, the autoimmune paradigm of vitiligo has long emphasized the coordinated interplay between resident cutaneous cells and infiltrating immune populations within the skin [2, 3]. In contemporary medicine, vitiligo is defined as a chronic, multifactorial, and polygenic disorder characterized by the appearance of achromic or hypopigmented macules on the skin and or mucous membranes, resulting from immune mediated destruction of melanocytes, largely driven by autoreactive T cells at the dermoepidermal interface [4] (Fig. 1). This framework has provided a robust basis for understanding vitiligo pathogenesis and for developing therapies aimed at controlling immune-mediated melanocyte loss. Within this paradigm, effective therapeutic strategies should not only attenuate cytotoxic responses targeting melanocytes but also re-establish cutaneous homeostasis through durable immune regulatory control of inflammation [5, 6].

Resolution of inflammatory processes is recognized as an active and highly regulated biological program, rather than a passive decline of pro-inflammatory signals. It involves specialized pro-resolving mediators, coordinated cellular reprogramming, and tissue repair mechanisms that collectively terminate inflammation and restore homeostasis [7–9]. However, the application of the concept of immune resolution to chronic, antigen-driven autoimmune diseases such as vitiligo requires careful conceptual refinement. In classical immunology, resolution refers to the active termination of inflammation following clearance of a transient stimulus, involving coordinated processes such as efferocytosis, lipid mediator class switching, and suppression of leukocyte recruitment. By contrast, autoimmunity is sustained by persistent autoantigen recognition and long-lived immune memory, representing a fundamentally distinct biological context [10].

Notably, recent advances have expanded the concept of resolution to include a post-resolution phase characterized by persistent immune remodeling, tissue-adapted immunity, and the accumulation of memory lymphocytes that shape subsequent immune responses. In this phase, tissues do not simply return to baseline but instead acquire a state of “adapted homeostasis,” which may be protective or, when dysregulated, may predispose to chronic inflammation and autoimmunity [11, 12]. Accordingly, defective resolution in chronic diseases should not be interpreted as a failure to terminate an acute inflammatory response, but rather as an inability to appropriately regulate and reprogram immune responses over time.

In vitiligo, disease persistence is primarily driven by melanocyte-specific tissue-resident memory T cells (T_RM_), which are maintained locally through interleukin (IL)-15-dependent survival signals and ongoing antigen presentation [13]. These cells establish a self-sustaining inflammatory niche in the skin that persists independently of the initial triggering events. Within this context, we propose that vitiligo reflects a failure of immune resolution in an expanded sense; namely, the inability to restrain pathogenic immune memory, re-establish immune tolerance, and restore a tissue environment permissive to melanocyte regeneration. This interpretation does not imply a classical failure to resolve inflammation after pathogen clearance, but rather a defect in the long-term regulatory mechanisms that normally constrain, compartmentalize, and ultimately silence autoreactive immune responses. Therefore, this distinction reconciles the concept of immune resolution with the antigen-driven nature of autoimmunity while preserving its relevance as a framework for understanding disease chronicity and guiding the development of targeted therapeutic strategies.

In this context, current treatments to vitiligo often achieve disease stabilization but rarely induce sustained long-term remission or skin repigmentation. This limitation likely reflects an incomplete targeting of the underlying pathogenic circuitry, which encompasses not only persistent immune activation but also defective regulation of immune memory and impaired tissue repair mechanisms. Hence, optimal therapeutic strategies should represent a realistic pathway toward long-term disease modification.

Advances in this field are expected to provide the foundation for precision-oriented interventions capable of limiting disease progression while improving repigmentation outcomes, which ultimately depend on the restoration of melanocyte regenerative capacity within the skin. In parallel, computational approaches, including machine learning-based analytical frameworks, are increasingly contributing to the identification of immune signatures and predictive biomarkers relevant to therapeutic stratification in vitiligo [14].

Considering this evolving landscape, a detailed appraisal of the mechanisms underlying vitiligo immunopathogenesis is essential to define the conceptual basis of emerging therapeutic platforms and to delineate how advances in disease immunobiology, together with technological innovation, are enabling increasingly targeted and rational approaches to immune modulation and tissue repair. Accordingly, this review does not aim to provide a comprehensive clinical overview, but rather to dissect the immunological and technological pipeline driving next-generation precision immunoengineering strategies for vitiligo. Importantly, this perspective builds on the recognition that vitiligo should not be viewed solely as a consequence of immune activation, but rather as a condition sustained by defective regulation of immune memory and impaired tissue regeneration. By targeting these interconnected processes, such approaches may enable durable vitiligo control without continuous immunosuppression, representing a realistic pathway toward long-term disease modification.

## Initiation of Vitiligo: Oxidative Stress, Early Immune Triggers and Innate Sensing

Research in vitiligo has progressively incorporated multiple pathogenic pathways, including biochemical, neural, and autoimmune processes, as well as additional mechanisms such as oxidative stress, microvascular alterations, defects in melanocyte adhesion, and genetic susceptibility. Collectively, these mechanisms converge into a unified pathogenic framework in which melanocyte-intrinsic stress responses interact with immune dysregulation to initiate and sustain disease within a genetically permissive context [15, 16].

**Fig. 2:**
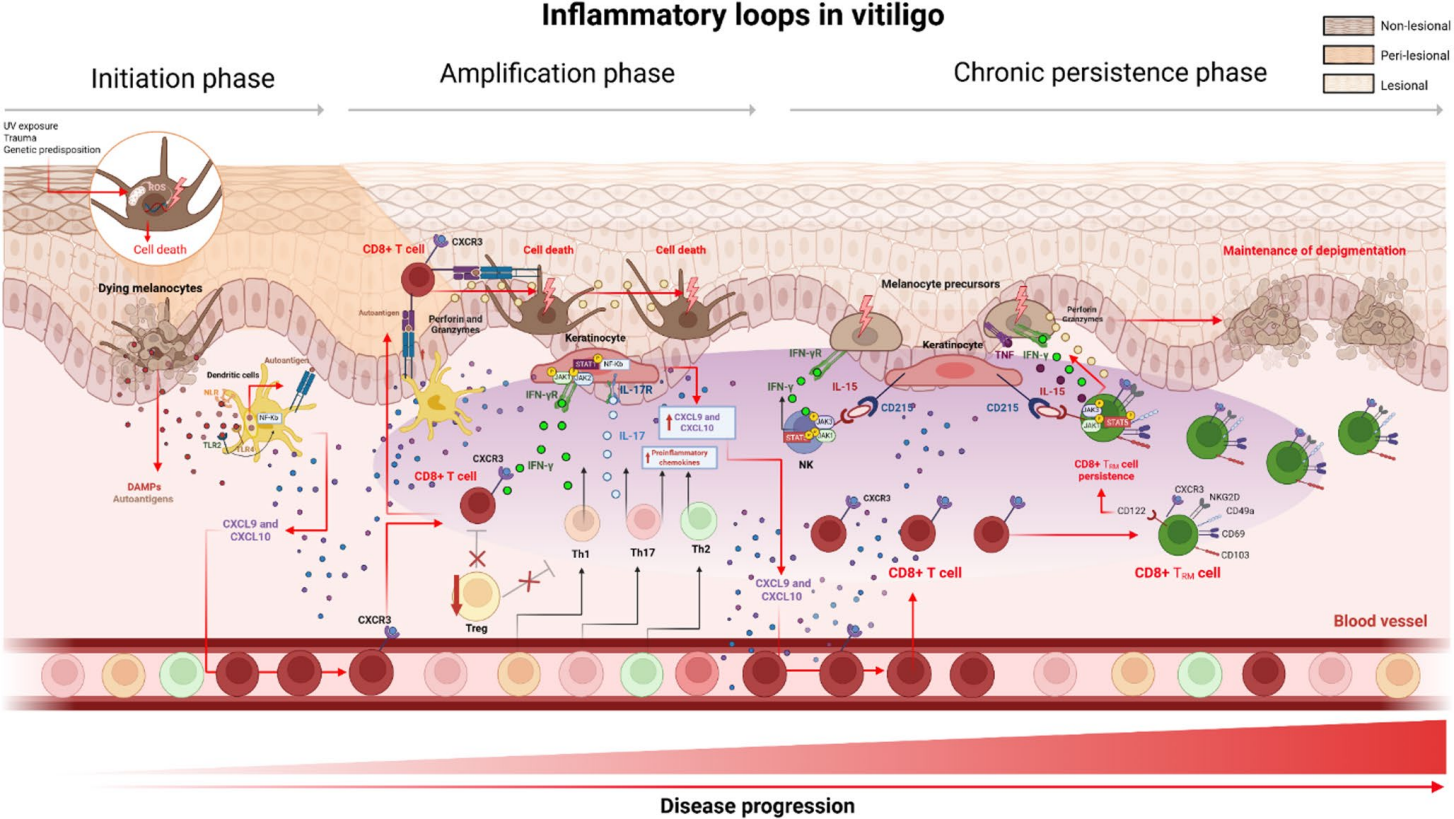
Inflammatory loops in vitiligo. Vitiligo is initiated by an autoimmune inflammatory process in the skin, in which UV exposure, trauma, and genetic susceptibility induce oxidative stress in melanocytes. Mitochondrial dysfunction leads to ROS generation, DNA damage, and melanocyte death, with subsequent release of DAMPs (HSP70, HMGB1) and melanocytic autoantigens. These signals activate dendritic cells via TLR and NLR pathways, promoting NF-κB dependent production of CXCL9 and CXCL10 and recruitment of CXCR3⁺ cytotoxic CD8⁺ T cells. In parallel, antigen presentation by dendritic cells activates CD8⁺ T cells, which mediate melanocyte killing through perforin and granzymes and secrete IFN-γ, amplifying inflammation through a positive feedback loop. Th1, Th2, and Th17 responses cooperate to sustain cutaneous inflammation, with IL-17 and IFN-γ enhancing chemokine production by keratinocytes, meanwhile the function of Tregs is impaired in vitiligo. As the disease progresses, CD8⁺ tissue-resident memory T cells persist in the skin in an IL-15-dependent manner and maintain depigmentation by promoting oxidative stress and damage to melanocyte precursors. Abbreviations: UV, ultraviolet radiation; ROS, reactive oxygen species; DAMPs, damage-associated molecular patterns; HSP70, heat shock protein 70; HMGB1, high mobility group box 1; PMEL, premelanosome protein; TLR, toll-like receptor; NF-κB, nuclear factor kappa B; CXCL, C-X-C motif ligand; CD, cluster of differentiation; CXCR3, C-X-C motif receptor 3; IFN-γ, interferon-γ; Th1, T helper 1; Th2, T helper 2; Th17, T helper 17; IL, interleukin; Treg, regulatory T cell; JAK, Janus kinase; STAT, signal transducer and activator of transcription; ILC1, innate lymphoid cells type 1; IFN-γR, interferon-γ receptor; NKG2D, Natural Killer Group 2, Member D; TNF, Tumor necrosis factor; TNFR, Tumor necrosis factor receptor; T_RM_, tissue-resident memory T cells. Created in BioRender

Intrinsic defects within melanocytes appear to contribute to their dysfunction, death, and subsequent depigmentation in vitiligo [17]. Importantly, melanocytes should not be viewed solely as passive targets, but as active participants in immune regulation, capable of modulating antigen presentation, cytokine production, and local immune interactions under stress conditions [18, 19]. These observations are supported by reduced catalase activity, an enzyme essential for neutralizing reactive oxygen species (ROS), in the skin of patients with vitiligo [20]. Notably, although melanin is essential for skin repigmentation and provides photoprotection to surrounding keratinocytes, the melanogenic pathway inherently generates substantial amounts of ROS, which can directly interfere with melanogenesis. In patients with vitiligo, oxidative stress induces significant alterations in the conformation and function of tyrosinase (TYR), a key melanocyte enzyme, thereby impairing melanin synthesis and potentially enhancing the generation of neoantigenic determinants that promote immune recognition [21]. These findings position melanocytes at the intersection of metabolic stress and immune activation in vitiligo. As a result, these cells occupy a particularly vulnerable position, since the very process required to restore pigmentation concurrently amplifies oxidative stress, further increasing their susceptibility to damage [22] (Fig. 2).

Beyond its effects on melanogenesis, hydrogen peroxide-mediated oxidative stress enhances melanocyte immunogenicity, in part through calreticulin translocation to the cell surface, which functions as a danger-associated signal promoting immune recognition. While this process contributes to local inflammatory activation, it primarily increases melanocyte vulnerability to immune-mediated damage, ultimately leading to the activation of pro-apoptotic pathways [23].

Necroptosis has also been evidenced by the accumulation of phosphorylated mixed lineage kinase domain-like protein (MLKL) and receptor-interacting protein kinase 3 (RIPK3) in melanocytes. Activation of this pathway promotes the release of damage-associated molecular patterns (DAMPs), which amplify local inflammation [24] and act as endogenous danger signals capable of alerting and activating immune cells [25]. The recognition of nucleic acids and other DAMPs is mediated by pattern-recognition receptors (PRRs), particularly Toll-like receptors (TLRs) and NOD-like receptors (NLRs), which are broadly expressed in innate immune cells, melanocytes and keratinocytes [26–28] (Fig. 2).

Within this innate sensing landscape, dendritic cells (DCs) emerge as central orchestrators of the transition from danger recognition to loss of immune tolerance. Plasmacytoid DCs (pDCs) respond to nucleic acid-containing DAMPs through TLR7/9 and cytosolic sensing pathways, including cGAS-STING-associated signaling, leading to type I interferon (IFN) production and amplification of local inflammatory responses. In parallel, conventional DC (cDCs) subsets contribute to antigen processing and presentation, with cDC1 preferentially mediating cross-presentation of melanocyte-derived antigens via major histocompatibility complex (MHC) class I, while cDC2 subsets shape local inflammatory polarization progression [29–33]. Through these coordinated functions, DC populations translate innate danger signals into sustained immune activation and facilitate the initiation of autoreactive responses in vitiligo, consistent with evidence that PRR-driven activation of innate immune cells, including dendritic cells, is a key early event in disease pathogenesis.

Indeed, among these DAMPs, high-mobility group box 1 (HMGB1) and heat shock protein 70 (HSP70) are prominent molecules released by stressed melanocytes and sensed by PRRs, thereby promoting inflammatory signaling and potentially contributing to the initiation of anti-melanocyte immunity in vitiligo [31]. Extracellular HMGB1 activates TLR2 and TLR4, thereby promoting nuclear factorkappa B (NF-κB)-dependent transcriptional responses [34]. In addition, the janus kinase (JAK)/signal transducer and activator of transcription (STAT)-1 pathway, a central downstream mediator of multiple pro-inflammatory cytokine receptors, has been implicated in regulating HMGB1 nuclear mobility and activity. STAT1-driven hyperacetylation promotes HMGB1 translocation from the nucleus to the cytoplasm, thereby linking inflammatory cytokine signaling to the release of endogenous danger signals and amplification of innate immune activation [32].

Similarly, inducible HSP70 (HSP70i), whose expression is influenced by inflammatory pathways such as JAK/STAT signaling, plays a critical role in coupling cellular stress to immune activation in vitiligo [33] (Fig. 2). In an animal model, overexpression of HSP70i markedly exacerbates depigmentation, while promoting dendritic cell activation and skewing antigen-presenting cells toward a pro-inflammatory phenotype [34, 35]. Soluble HSP70i further enhances antigen uptake, processing, and presentation, thereby amplifying autoreactive immune responses Through this altered metabolic and secretory profile, specific keratinocyte subsets contribute to the persistence of immune activation and the maintenance of depigmented lesions [31]. Collectively, these findings position HMGB1 and HSP70i at the intersection of oxidative stress, innate immune sensing, and cytokine-driven amplification, highlighting their role not only as passive markers of cellular damage but as active mediators of immune activation.

Keratinocytes also function as active sentinels of tissue stress, through cytosolic receptors such as melanoma differentiation-associated protein 5 (MDA5), which sense damage signals and lead to enhanced production of chemokine (C-X-C motif) ligand (CXCL) 10 and CXCL16 for T cell recruitment [35–37]. Furthermore, inflammasome activation in keratinocytes regulates IL-1β production and sustains inflammatory signaling within the skin microenvironment [38].

Regarding the innate lymphoid cells, particularly type 1 populations such as natural killer (NK) cells and ILC1, it is known that they play a critical role in the early stages of melanocyte destruction in vitiligo. These cells are enriched in the blood and non-lesional skin of patients and exhibit increased production of IFN-γ, a key cytokine that induces CXC motif chemokine receptor 3 (CXCR3B) expression in melanocytes, rendering them more susceptible to apoptosis and immune-mediated damage [22]. In addition, NK cells amplify local inflammation through the secretion of cytokines and chemokines that promote the recruitment and activation of additional immune cell subsets, thus enhancing melanocyte vulnerability to death [22] (Fig. 2). In fact, experimental evidence further supports a direct cytotoxic role for NK cells in vitiligo, demonstrating their capacity to mediate melanocyte killing and initiate early inflammatory responses. Consistently, human ILC1 populations accumulate in inflamed tissues and produce IFN-γ, reinforcing local immune activation and facilitating the transition from innate to adaptive immune responses [39].

Finally, extrinsic factors such as gut dysbiosis may also contribute to the initiation phase by modulating oxidative stress and systemic immune tone. In a murine model of vitiligo, the microbiota-derived metabolite hippuric acid promoted ROS accumulation in melanocytes, whereas microbiota depletion or probiotic administration reduced oxidative burden and suppressed depigmentation. Elevated serum levels of hippuric acid have also been detected in patients with vitiligo, suggesting a potential link between microbial metabolism and early disease triggers [40]. Altogether, these findings reinforce the concept that oxidative stress in vitiligo is not merely a cell-intrinsic event, but is shaped by systemic metabolic and environmental cues that can amplify redox imbalance and prime the immune system.

## Immunometabolic Dysfunction Linking Oxidative Stress to Immune Activation

While oxidative stress initiates melanocyte damage, accumulating evidence indicates that its pathogenic effects are largely mediated through immunometabolic reprogramming, which acts as a central interface linking cellular stress to sustained immune activation [41–43].

At the cellular level, melanocytes exposed to oxidative stress exhibit mitochondrial dysfunction, characterized by altered membrane potential, impaired oxidative phosphorylation, and increased production of reactive oxygen species. Sublethal oxidative stress can induce oligomerization of voltage-dependent anion channel 1 (VDAC1), leading to the release of mitochondrial DNA (mtDNA) into the cytosol and subsequent activation of the cGAS-STING pathway. This process promotes innate immune activation and type I IFN responses, thereby linking metabolic stress to inflammatory signaling [41]. This pathway represents a critical mechanistic bridge between metabolic dysfunction and innate immune sensing, functionally extending the early stress signals described above into sustained immune activation programs.

In parallel, disruption of redox homeostasis is exacerbated by impaired activation of the nuclear factor erythroid 2-related factor 2-antioxidant response element (Nrf2-ARE) pathway, resulting in decreased expression of antioxidant enzymes such as heme oxygenase-1 (HO-1) and reduced capacity to neutralize oxidative damage [44]. Beyond oxidative stress, ferroptosis, an iron-dependent form of lipid peroxidation-driven cell death, may contribute to melanocyte loss in vitiligo. Rather than representing an isolated mechanism of cell death, ferroptosis appears to arise from underlying immunometabolic dysfunction, linking redox imbalance and lipid peroxidation to enhanced cellular vulnerability and inflammatory signaling [43]. However, the causal role of ferroptosis in human vitiligo and its contribution to immune activation remain to be fully established.

Interestingly, metabolic dysfunction is not restricted to melanocytes but also affects other skin-resident cells. Keratinocytes and fibroblasts exposed to oxidative stress undergo metabolic reprogramming that enhances their pro-inflammatory secretory profile. In particular, mitochondrial stress-induced release of mtDNA in dermal fibroblasts (not only in melanocytes as described above) activates cGAS-STING signaling and promotes a senescence-associated secretory phenotype (SASP), characterized by increased production of cytokines and chemokines that support CD8⁺ T-cell recruitment and persistence [41, 45]. Furthermore, metabolic stress directly contributes to immune activation through inflammasome signaling. The triggering of NLRP3 inflammasome in keratinocytes promotes IL-1β production, which enhances T-cell activation and amplifies cutaneous inflammation [46, 47].

Collectively, these findings support a model in which immunometabolic dysfunction acts not merely as a consequence of oxidative stress, but as a central amplifier that sustains immune activation, reinforces cytokine signaling, and promotes the activation of autoreactive T cells within the skin. Importantly, persistent metabolic stress also may contribute to defective constrainment of immune response by maintaining low-grade inflammation, impairing regulatory mechanisms, and supporting the survival of memory T cells, thereby linking immunometabolic dysfunction to chronic disease progression and relapse.

## Immune Amplification Loops and the Transition to Chronic Autoimmunity

Rather than representing a strictly linear cascade, immune amplification in vitiligo is better conceptualized as a multilayered and spatially coordinated network. Early events driven by oxidative stress, cytosolic nucleic acid sensing, and innate immune activation establish a permissive inflammatory niche that licenses subsequent adaptive immune responses that result in different clinical outcomes.

In line with this, the distinction between active (progressive) and stable vitiligo reflects differences in the magnitude and persistence of these amplification circuits. Active disease is characterized by sustained and self-reinforcing inflammatory signaling, leading to ongoing melanocyte loss and lesion expansion. In contrast, stable vitiligo represents a state in which these amplification loops are partially constrained, allowing clinical stability despite the persistence of resident immune populations and subclinical inflammatory activity [48].

From this perspective, keratinocytes and innate lymphoid populations (besides melanocytes) act as key intermediaries, sustaining chemokine gradients and cytokine signaling both prior to, and independently of, fully established cytotoxic responses [39, 49]. Then, the adaptive immune dysregulation in vitiligo reflects a coordinated interplay between these cells and CD4⁺ as well as cytotoxic CD8⁺ T cells, whose functional imbalance drives melanocyte-targeted autoimmunity. Altogether, these populations operate within an integrated network in the skin microenvironment that links immune polarization, effector activation, and defective immune regulation.

CD4⁺ T cells contribute to disease pathogenesis by shaping the inflammatory milieu through distinct helper subsets, including Th1, Th2, Th17, and regulatory T cells (Tregs) [50–55]. Alterations in their frequency and function reinforce the autoimmune nature of vitiligo [50, 51]. Reduced frequencies of circulating effector and central memory Th1, Th17, and cytotoxic T-cell subsets in patients are consistent with their preferential migration to the skin, where they sustain local immune activation. Within this framework, CD4⁺ T cells act as key orchestrators of immune polarization, promoting cytokine environments that favor cytotoxic responses [56]. In fact, the effector phase of vitiligo is predominantly mediated by autoreactive CD8⁺ T cells that recognize melanocyte-derived autoantigens [57].

The antigenic repertoire in vitiligo involves melanocyte differentiation antigens, including PMEL/gp100 and Melan-A/MART vitiligo [58], but also multiple differentiation-associated proteins such as TYR, tyrosinase-related protein 1 (TYRP1/gp75), and tyrosinase-related protein 2 (TYRP2), all recognized by autoreactive CD8⁺ T cells. Several immunodominant epitopes have been characterized in the context of HLA class I molecules, particularly HLA-A*02:01. Among these, the Melan-A/MART1-derived peptide AAGIGILTV and the gp100-derived peptide IMDQVPFSV are well-established epitopes capable of eliciting cytotoxic responses in both vitiligo and melanoma. TYR-derived epitopes presented by HLA-A*02:01 and other class I alleles further support the concept of a multi-antigenic autoimmune response [58–61] (Table 1).

**Table 1:** Melanocyte-derived autoantigens, corresponding epitopes, and HLA restriction

Autoantigen	Epitopes	HLA restriction	Ref.
PMEL / gp100	• IMDQVPSFV; • YLEPGPVTV; • YLEPGPVTA.	HLA-A2; HLA-A2; HLA-A2.1.	[58, 62]
Melan-A / MART-1	• ELAGIGILTV; • EAAGIGILTV; • AAGIGILTV.	HLA-A*02:01; HLA-A*02:01; HLA-A2.	[58, 59, 63]
TYR	• YMDGTMSQV; • AFLPWHRLF; • AFLPWHRLFL.	HLA-A*02:01; HLA-24; HLA-24.	[59, 64]
TYRP1/gp75	Not defined.	HLA-A31.	[65]
TYRP-2/DCT	• LLGPGRPYR.	HLA-A31.	[66]
MC1R	• TILLGIFFL; • FLALIICNA; • AIIDPLIYA.	HLA-A2; HLA-A2; HLA-A2.	[67]

Abbreviations: TYR, tyrosinase, *PMEL* Premelanosome Protein, *gp100* Glicoprotein 100, *MART-1* Melanoma Antigen Recognized by T cells, *TYRP1* Tyrosinase-Related Protein 1, *gp75* Glicoprotein 75, *TYRP-2* Tyrosinase-Related Protein 2, *DCT* Dopachrome tautomerase, *MC1R* Melanocortin 1 Receptor

Notably, vitiligo may involve a dynamic process of antigenic diversification, or epitope spreading, in which initial immune responses expand to recognize additional melanocyte antigens over time [93, 94]. Evidence from melanoma patients treated with anti-programmed cell death 1 (PD-1) therapy supports the concept that T-cell responses can expand from initial targets to additional melanocyte antigens, illustrating a lineage-specific autoimmunity and epitope spreading in the skin. Mechanistically, ongoing melanocyte stress and death promote the release of autoantigens and cryptic epitopes, amplifying melanocyte-specific CD8⁺ T-cell responses and reinforcing tissue damage [95, 96]. This process establishes a feed-forward inflammatory circuit that sustains pathogenic T-cell responses and contributes to disease chronicity and relapse [97]. nnnnn.

In this framework, progressive diversification of antigenic targets provides a mechanistic link between early immune activation and defective immune regulation, as the expansion of antigen specificity increases the complexity of restoring antigen-specific tolerance and long-term immune quiescence in vitiligo.

Melanocytes themselves can also acquire antigen-presenting capacity under inflammatory conditions [98] (Fig. 2) and modulate the local immune microenvironment, besides actively regulating immune responses through the export of peptide-MHC complexes [19]. Thus, melanocytes should be considered active participants in shaping local immune responses, capable of influencing T-cell activation, tolerance, and potentially immune evasion mechanisms depending on the inflammatory context. In the context of vitiligo, dysregulation of these mechanisms could favor sustained activation of autoreactive CD8⁺ T cells, thereby contributing to persistent inflammation and impaired immune regulation.

In parallel, the recruitment and retention of autoreactive T cells in the skin are driven by chemokine gradients, particularly CXCL9, CXCL10, and CXCL11 produced by keratinocytes and other resident cells, establishing an inflammatory amplification circuit [99]. Among these, CXCL10 shows a strong correlation with disease activity and severity, highlighting its dual role as a pathogenic mediator and biomarker [100]. Binding of these chemokines to CXCR3 promotes both recruitment and retention of CD8⁺ T cells within the epidermis [101].

On the other hand, T_RM_ cells in vitiligo display functional heterogeneity, encompassing cytotoxic and cytokine-producing subsets that contribute to melanocyte-directed immune responses [99, 102, 103]. Among CD8^+^ T_RM_ play a central role in sustaining disease chronicity. These cells persist long-term within the epidermis, where they remain poised for rapid effector responses upon antigen re-encounter [56, 97]. In vitiligo, T_RM_ cells exhibit a cytotoxic and IFN-γ-producing phenotype, reinforced by IL-15-dependent survival signals provided by keratinocytes. This establishes a stable, self-sustaining inflammatory niche that underlies disease persistence and relapse (Fig. 2) [97, 101, 102, 104] (Fig. 2).

At the molecular level, IFN-γ signaling is mediated through IFNGR1/IFNGR2, leading to activation of JAK1/JAK2, STAT1 phosphorylation, and transcription of pro-inflammatory genes that drive recruitment and effector function of autoreactive T cells [105]. In addition to its immunomodulatory role, IFN-γ directly impairs melanocyte function by suppressing melanogenesis [106]. Experimental models further demonstrate that IFN-γ neutralization reduces depigmentation and T-cell accumulation, supporting its central role in disease pathogenesis [103]. In parallel, IL-15 is a key factor associated with disease severity [107]. This cytokine promotes the activation and survival of T cells and natural killer (NK) cells through JAK1- and JAK3-dependent signaling pathways, thereby regulating inflammatory effector functions. Importantly, IL-15 has been implicated in the maintenance of T_RM_ cells [108]. At the mechanistic level, IL-15 signaling depends on IL-15Rα (CD215), which binds IL-15 with high affinity and presents it in trans to neighboring T cells via the IL-2/15Rβ (CD122) and common γ-chain receptors. This trans-presentation mechanism supports T-cell activation, survival, and tissue retention [109]. In vitiligo lesions, keratinocytes overexpress CD215, enabling sustained IL-15 trans-presentation and supporting the persistence of pathogenic tissue-resident memory T-cell populations (Fig. 2) in both human disease and murine models during active inflammation [108].

Recent advances in T-cell receptor (TCR) repertoire and immune profiling analysis have provided aditional mechanistic insights into antigen-specific responses in vitiligo. High-throughput sequencing of the CD8⁺ TCRβ repertoire in the non-segmental disease has revealed reduced repertoire diversity, the presence of highly expanded clonotypes, and disease-associated TRBV/TRBJ usage patterns, supporting antigen-driven clonal selection in these patients [110]. Complementary phenotypic analyses of T_RM_ cells have further demonstrated the accumulation of polyclonal, antigen-experienced CD8⁺ T-cell populations enriched in lesional and perilesional skin, consistent with ongoing antigen-driven selection and local expansion of autoreactive clones [97, 101]. These clonotypes frequently display a tissue-resident memory phenotype (CD69⁺CD103⁺) and are enriched for IFN-γ production and cytotoxic effector functions, supporting their role as an antigen-experienced population central to melanocyte destruction [97, 101]. Moreover, the TCR sequencing analyses in melanoma patients developing vitiligo under anti-PD-1 therapy revealed a distinct clonal architecture across compartments, with differential clonotype distribution between peripheral blood, primary tumors, metastases, and vitiligo lesions, and a preferential sharing of TCR sequences between vitiligo and metastatic tissues rather than with circulating T cells [111]. Together, these findings support a model in which antigen-driven clonal expansion occurs preferentially within the skin, giving rise to a stable pool of autoreactive T_RM_ cells that sustain local immune responses with limited reliance on continuous recruitment from the circulation. While some degree of clonal overlap across compartments exists, the overall evidence favors the concept that immune compartmentalization in the skin is a central feature of vitiligo pathogenesis, underpinning both disease persistence and site-specific recurrence.

A further study showed the coexistence of active effector programs and regulatory constraints across distinct skin compartments in vitiligo, reflecting a dynamic balance between antigen-driven T cell activation and local immunoregulatory mechanisms [112]. These findings indicate a complex balance between effector function and regulatory restraint within the lesional microenvironment.

Therefore, the persistence of clonally expanded, antigen-specific T-cell populations, together with the inability of regulatory pathways to adequately restrain these responses, emerges as a critical determinant of disease persistence.

Within this context, a critical unresolved question concerns the origin, diversification, and persistence of antigenic stimuli capable of sustaining autoreactive T-cell responses over time. While endogenous melanocyte-derived antigens constitute primary targets, increasing evidence suggests that exogenous factors, particularly the microbiota, may contribute to shaping and expanding the antigenic repertoire in vitiligo.

As described before, there is a significant association between gut dysbiosis and altered microbial metabolite profiles in vitiligo patients, including reduced microbial diversity and shifts in taxonomic composition [113, 114]. Beyond metabolic alterations, an emerging and mechanistically relevant question is whether microbiota-derived antigens may directly contribute to disease initiation or persistence through molecular mimicry. This phenomenon, defined as structural or sequence similarity between microbial peptides and self-antigens, has been implicated in several autoimmune diseases and may enable cross-reactive T-cell responses that break immune tolerance [115, 116].

In this context, commensal anaerobic bacteria, including *Bacteroides fragilis*, have been reported to express peptides capable of mimicking host antigens and inducing autoreactive immune responses in experimental and clinical settings [117, 118]. A paradigmatic example is provided by Sjögren’s syndrome, in which microbial peptides derived from *B. fragilis* can activate autoreactive T cells targeting the Ro60 autoantigen [117]. Although direct evidence of analogous mechanisms in vitiligo is currently lacking, recent studies have reported an increased relative abundance of *B. fragilis* in patients with nonsegmental vitiligo compared with healthy controls, suggesting a potential role for specific microbial taxa in disease-associated immune modulation [119].

Notably, not only at the gut, but skin microbiota profiles are also altered in vitiligo, with distinct bacterial signatures in lesional skin associated with metabolic and mitochondrial dysfunction [114]. Within this expanded antigenic framework, it is plausible that microbial antigens sharing structural similarity with melanocyte-derived proteins, such as PMEL, TYR, or MART-1, may contribute to the priming or diversification of cross-reactive T-cell responses in genetically susceptible individuals. Once initiated, these responses may be further amplified locally in the skin through antigen presentation, epitope spreading, and sustained T_RM_ persistence, thereby reinforcing clonal expansion and contributing to chronic inflammation and failure of immune resolution. However, direct evidence supporting molecular mimicry between microbial and melanocyte-derived epitopes in human vitiligo remains lacking. Nevertheless, this hypothesis is consistent with established mechanisms in other autoimmune diseases and represents a promising avenue for future investigation in vitiligo.

Overall, the potential progressive diversification of antigenic targets further reinforces the transition from acute immune activation to chronic autoimmunity, linking immune amplification to failure of immune regulation. Within this framework, the persistence of metabolic and innate immune activation, together with the sustained presence of T_RM_ cells, indicates that disease chronicity is not solely driven by ongoing immune stimulation but also by a failure to engage effective mechanisms to constrain dysregulated responses.

Apart from T-cell memory, humoral immune memory may also play a role in vitiligo persistence. Autoantibodies targeting melanocyte antigens such as TYR and TYRP-2 have been consistently detected in patients with non-segmental vitiligo, whereas they are largely absent in segmental vitiligo, which also exhibits reduced plasmablast frequencies, indicating a diminished humoral response [120, 121]. In parallel, active vitiligo is characterized by a shift in B cell compartments, with reduced naive B cells and expansion of memory and class-switched B cells, reflecting enhanced germinal center activity [122]. This process is supported by increased frequencies of T follicular helper (Tfh) cells, which correlate with disease severity and progression, including body surface involvement and spreading activity [123]. At the transcriptional level, progressive disease is associated with upregulation of IgG heavy chain genes in memory B cells, consistent with antigen-experienced, class-switched populations [32]. Altogether, these findings indicate that a mature and antigen-driven humoral immune response is present in active vitiligo and could contribute to disease amplification. Nevertheless, current evidence supports a secondary and comparatively minor role for B cells and autoantibodies, which likely act as ancillary modulators of immune activation, whereas cytotoxic T cell responses remain the predominant drivers of melanocyte destruction.

In parallel, innate immune memory may also contribute to chronic inflammatory responses. In humans, NK cells can acquire memory-like properties characterized by enhanced IFN-γ production and increased responsiveness upon [124]. Considering vitiligo, NK cells exhibit heightened sensitivity to oxidative stress and DAMPs, along with increased IFN-γ production, supporting limited but indirect evidences of trained-like phenotype of innate activation [39]. These findings are consistent with a model in which NK cells may amplify type 1 immune responses and contribute to the maintenance of local inflammation.

Together, these findings indicate that vitiligo persistence is sustained by multilayered immune memory, integrating tissue-resident, circulating, humoral, and potentially innate compartments. This distributed memory architecture provides a mechanistic explanation for disease relapse following apparent clinical stability, as autoreactive immune cells may persist outside clinically active lesions and rapidly reinitiate pathogenic responses upon exposure to local or systemic triggers.

This persistent and spatially distributed immune memory represents a major barrier to effective immune regulation and long-term disease control. Rather than undergoing contraction and restoration of immune homeostasis, autoreactive populations are continuously maintained and reactivated across compartments, preventing the termination of inflammatory circuits. From this perspective, vitiligo can be understood not only as a disease of sustained immune activation but also as a condition characterized by defective regulation of pathogenic immune responses and incomplete resolution of inflammation.

These concepts provide a mechanistic basis for understanding how persistent immune memory, impaired regulatory networks, and altered tissue adaptation collectively contribute to disease chronicity in vitiligo. In addition, they offer a critical foundation for the development of precision therapeutic strategies, in which interventions can be tailored according to disease stage, immune phenotype, and the underlying pathogenic mechanisms driving melanocyte loss in individual patients.

## Failure of Immune Regulatory Control and Mechanisms Sustaining Chronic Inflammation

The maintenance of immune tolerance is essential for preventing autoimmunity, and defects affecting both central and peripheral tolerance checkpoints are increasingly recognized as key contributors to vitiligo pathogenesis. These mechanisms operate in a hierarchical and integrated manner, beginning with central tolerance in primary lymphoid organs and extending to peripheral regulatory networks that actively restrain autoreactive responses.

Central tolerance is primarily established in the thymus, where developing T cells undergo stringent selection processes. A critical component of this process is the autoimmune regulator (AIRE), which drives the ectopic expression of tissue-specific antigens in medullary thymic epithelial cells, enabling the negative selection of autoreactive T-cell clones [125]. Functional impairment or genetic variation in AIRE disrupts this deletional process and compromises the generation of thymus-derived regulatory T cells (Tregs), thereby permitting the escape of autoreactive lymphocytes into the periphery. In this context, a specific AIRE single-nucleotide polymorphism (CGCC haplotype) has been associated with increased susceptibility to vitiligo, supporting a role for defective central tolerance in disease initiation [126].

While central tolerance establishes a foundational layer of immune self-recognition, peripheral tolerance mechanisms are required to continuously control autoreactive cells that evade thymic deletion. Among these, Tregs play a pivotal role. Defined by the expression of CD4, CD25 (IL-2Rα), and the transcription factor FOXP3, Tregs suppress autoreactive CD4⁺ and CD8⁺ T cells and maintain immune homeostasis under both physiological and inflammatory conditions [127]. Their function is tightly linked to IL-2 signaling and immune checkpoint pathways.

Genetic and functional alterations affecting key Treg-associated molecules further compromise peripheral tolerance in vitiligo. Polymorphisms in IL-2 receptor alpha chain (IL2RA) and cytotoxic T-lymphocyte associated protein 4 (CTLA-4), which are genes encoding molecules critical for Treg stability and suppressive function, have been associated with increased disease susceptibility to vitiligo [128]. IL-2Rα (CD25) contributes to the high-affinity IL-2 receptor complex and limits effector T-cell expansion by IL-2 sequestration, whereas CTLA-4 acts as a central immune checkpoint that attenuates T-cell activation through competitive inhibition of costimulatory signaling [129, 130].

The PD-1/PD-L1 axis represents an additional checkpoint involved in the resolution of inflammation and induction of T-cell exhaustion. Dysregulation of PD-1 signaling has been observed in vitiligo, with increased PD-1 expression on CD8⁺ T cells in non-lesional skin and enrichment of PD-1⁺ Tregs and CD8⁺ T cells in repigmented areas, suggesting a dynamic and context-dependent role in modulating immune responses during disease progression and recovery [112]. Consistent with this regulatory function, immune checkpoint blockade targeting in oncology is strongly associated with the development of vitiligo-like depigmentation, reflecting a breakdown of peripheral tolerance to melanocyte antigens and enhanced cytotoxic T-cell activity [111, 131]. These clinical observations reinforce the importance of intact checkpoint signaling in maintaining immune homeostasis in the skin [132].

Beyond genetic susceptibility, quantitative and qualitative defects in Tregs are consistently observed in vitiligo. Patients exhibit reduced frequencies of circulating and skin-resident Tregs, accompanied by impaired suppressive capacity [54, 133–135]. Experimental evidence supports a causal relationship, as adoptive transfer of Tregs in vitiligo-prone transgenic models induces sustained disease remission [136]. Mechanistically, Tregs in vitiligo may acquire a dysfunctional phenotype characterized by reduced FOXP3 and NFATc1 expression, diminished production of IL-10 and transforming growth factor (TGF)-β, and a shift toward Th1-like pro-inflammatory features. This functional instability results in inadequate control of effector T cells and increased IFN-γ production, which directly contributes to melanocyte destruction [137].

Importantly, spatial regulation of Treg activity within the skin also appears to be disrupted. Single-cell transcriptomic analyses have identified a CCL5-CCR5 axis that governs Treg positioning and enables suppression of CD8⁺ T cells through a shared chemokine circuit. Disruption of this axis may impair local immune regulation, further facilitating or amplifying tissue-specific autoimmunity [138]. These findings converge on the concept that vitiligo results not only from defects in tolerance induction but also from a failure to regulate inadequate responses, driven by the combined breakdown of Treg-mediated suppression and immune checkpoint pathways. This integrated framework provides a strong rationale for therapeutic strategies that extend beyond conventional immunosuppression, aiming instead to restore immune tolerance and promote tissue repair.

**Fig. 3:**
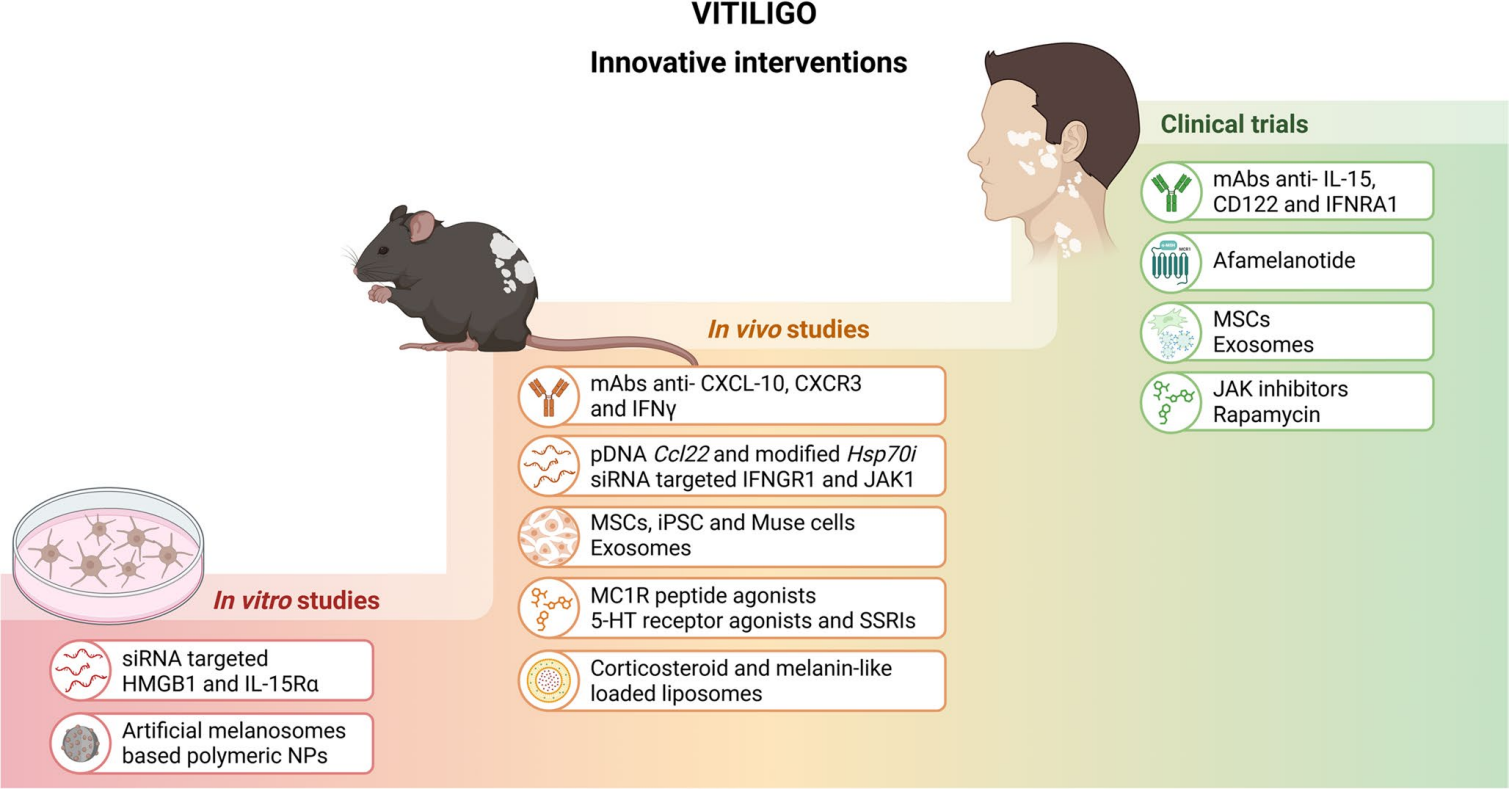
Technology-driven advanced therapeutic interventions for vitiligo. Overview at hierarchical levels of preclinical and clinical stages of advanced therapeutic strategies. Interventions targeting the active phase of the disease and immune dysregulation include approaches that block cytokine signaling, T cell recruitment and activation, tissue-resident memory T cells, and pathways related to oxidative stress. Regenerative and immunomodulatory strategies comprise extracellular vesicles, induced mesenchymal and pluripotent stem cells, and melanogenic pathway agonists. Advanced delivery modalities, particularly nanoparticle-based platforms, can support these interventions by improving skin penetration, promoting tissue-targeted delivery, and increasing the intracellular availability of biologics, nucleic acids, and small molecule therapeutics used alone or in combination at different stages of vitiligo development. Abbreviations: siRNA, small interfering RNA; pDNA, plasmid DNA; CXCL, C-X-C motif chemokine ligand; CD, cluster of differentiation; CXCR3, C-X-C motif chemokine receptor 3; IFNγ, interferon gamma; JAK, Janus kinase; IFNRA1, interferon receptor subunit 1; HMGB1, High-Mobility Group Box 1; IL-15Rα, interleukin-15 receptor subunit alpha; 5-HT, serotonin receptor; SSRI, selective serotonin reuptake inhibitors; MC1R, melanocortin receptor 1; HSP70i, 70 kDa inducible heat shock protein; IFNGR1, interferon gamma receptor 1; NP, nanoparticle; MSC, mesenchymal stem cells; iPSC, induced pluripotent stem cells. Created in BioRender

Within this perspective, emerging therapies are increasingly designed to reestablish regulatory networks and recalibrate immune homeostasis. The following section therefore examines how both established (Table 2) and novel therapeutic approaches (Fig. 3) target these interconnected mechanisms. By bridging traditional immunosuppressive modalities with innovative strategies grounded in immunological engineering, recent advances illustrate a paradigm shift toward more precise, mechanism-driven interventions in the management of vitiligo.

**Table 2:** Therapeutic strategies currently used in clinical practice

Strategy	Drug/ procedure	Main target	Main effect on vitiligo	Type of vitiligo evaluated	Ref.
Phototherapy	UVB-NB light	Immune cells and melanocytes	Immunomodulation and repigmentation	Nonsegmental vitiligo (focal, acrofacial, and generalized subtypes)	[68, 69]
Glucocorticoids (steroidal anti-inflammatory and immunosuppressive agents)	Betamethasone, dexamethasone, clobetasol, mometasone, prednisolone	Imumne cells	Immunomodulation	Segmental and nonsegmental vitiligo (progressive or unstable/stable*)	[70–72]
Glucocorticoids plus photo or laser therapy	-	Immune cells and melanocytes	Immunomodulation and repigmentation	Nonsegmental vitiligo	[73, 74]
Calcineurin inhibitors (immunomodulatory macrolactams; small molecules)	Tacrolimus	T cells	Immunomodulation	Nonsegmental vitiligo (acrofacial subtype)	[75, 76]
Calcineurin inhibitors plus photo or laser therapy	-	Immune cells and melanocytes	Immunomodulation and repigmentation	Nonsegmental vitiligo	[77–79]
JAK-STAT pathway inhibitors; immunomodulatory small molecules	Ruxolitinib^a^, baricitinib, tofacitinib	Immune cells	Immunomodulation	Nonsegmental vitiligo	[80, 81]
JAK inhibitors plus photo or laser therapy	-	Immune cells and melanocytes	Immunomodulation and repigmentation	Nonsegmental vitiligo (progressive or stable)	[82–85]
Cell therapy	Split-thickness skin grafts, suction blister grafts, and punch grafts (with or without in vitro expansion)	Epidermal-follicular cells and melanocytes	Repigmentation	Segmental and nonsegmental vitiligo (stable).	[86–88]
Cell therapy plus photo or laser therapy	-	Immune cells, epidermal-follicular cells and melanocytes	Immunomodulation and repigmentation	Segmental and nonsegmental vitiligo (stable).	[89–91]

Abbreviations: *NB-UVB* Narrowband ultravioletB light, *JAK* Janus kinases, *STAT* Signal Transducers and Activators of Transcription

^a^ Drug (Opzelura^®^, ruxolitinib 1.5% cream) approved for nonsegmental vitiligo by the U.S. Food and Drug Administration (2022) and the European Medicines Agency (2023)

*Progressive vitiligo is characterized by the rapid spread of lesions or the appearance of new lesions within 3 months, while stable vitiligo presents a 12-month period of stability in the appearance or progression of lesions [92]

## Traditional Approaches and Innovative Therapeutic Engineering in the Management of Vitiligo

### From Broad Immunosuppression to the Limits of Immune Resolution

For several decades, corticosteroids have been widely used in the management of active vitiligo due to their potent anti inflammatory and immunosuppressive properties. At the molecular level, these agents broadly inhibit inflammatory signaling through genomic and non-genomic mechanisms, including suppression of transcription factors such as NFκB and AP1, inhibition of eicosanoid synthesis, and induction of anti inflammatory mediators that attenuate innate and adaptive immune activation [139]. These effects result in reduced cytokine production and transient attenuation of T-cell activity within the skin. Clinically, topical and systemic corticosteroids are employed to stabilize disease activity and, in selected cases, induce partial repigmentation, with systemic regimens such as oral mini pulse protocols reserved for rapidly progressive disease [140].

However, despite effectively suppressing inflammation, corticosteroids do not specifically target key pathogenic circuits underlying vitiligo. In particular, they fail to selectively disrupt the IFN-γ/JAK/STAT axis and its downstream induction of CXCL9 and CXCL10, which are central to the recruitment and retention of CXCR3⁺ autoreactive CD8⁺ T cells in the epidermis [141–143]. Moreover, these agents do not eliminate or functionally reprogram T_RM_ cells, which persist in the skin and retain the capacity to rapidly reinitiate cytotoxic responses upon treatment withdrawal. As a result, corticosteroid therapy is frequently associated with disease relapse, reflecting suppression of immune activity rather than restoration of immune tolerance or resolution of pathogenic memory responses [144, 145].

Calcineurin inhibitors, including tacrolimus and pimecrolimus, act through a more targeted mechanism by blocking calcineurin-dependent dephosphorylation of NFAT, thereby inhibiting transcription of T cell-derived cytokines. These agents are commonly used as topical therapies, particularly for sensitive anatomical areas, and play an important role in maintenance strategies due to their favorable safety profile [146, 147]. This results in reduced T-cell activation and cytokine production, particularly within the CD4⁺ compartment. Clinically, these agents are widely used as topical therapies, especially in sensitive areas, and demonstrate efficacy in inducing repigmentation, particularly when combined with phototherapy [75–79, 148, 149].

Nevertheless, similar to corticosteroids, calcineurin inhibitors do not adequately interfere specifically with the recruitment of autoreactive CD8⁺ T cells or the survival signals sustaining T_RM_ populations, such as IL-15 trans-presentation within the epidermal niche [108, 150]. Consequently, their effects are largely limited to modulation of active inflammation, without promoting durable immune reprogramming or preventing disease recurrence [151].

Within this broadly immunosuppressive framework, additional pharmacological agents have been explored as adjunctive therapies. Topical 5-fluorouracil when combined with lasers or microneedling appears superior efficacy in controlling disease progression and inducing repigmentation, particularly in acral areas and over bony prominences (e.g., elbows and knees) [152–155]. Similarly, methotrexate has been reported to improve clinical outcomes and repigmentation when used in combination with oral mini-pulse dexamethasone in cases of advanced and unstable vitiligo [156, 157]. In addition, low doses of oral methotrexate have been shown to prevent progression and allow for better and faster repigmentation in focal vitiligo lesions compared to phototherapy alone [158]. Mechanistically, methotrexate inhibits folate-dependent metabolic pathways and exerts anti-inflammatory effects partly through increased adenosine signaling; however, its impact on pathogenic T-cell subsets and resident immune memory in vitiligo remains insufficiently characterized in primary experimental studies [159]. While such approaches may improve disease control in specific contexts, they predominantly rely on generalized immune suppression.

Rapamycin represents a further example of a repurposed immunomodulatory agent with pleiotropic effects. By inhibiting mTOR signaling, rapamycin can modulate T-cell differentiation and promote autophagy, while also enhancing melanocyte function through upregulation of microphthalmia-associated transcription factor (MITF) and melanogenic enzymes [5, 160]. Based on these findings, topical rapamycin is currently under clinical investigation for vitiligo (NCT05342519). Nonetheless, its therapeutic rationale remains centered on immune and metabolic modulation rather than induction of sustained immune resolution.

Overall, these classical, adjunctive, and repurposed therapies underscore the conceptual limitations of strategies focused predominantly on broad immune suppression. Although they may stabilize disease activity and enhance repigmentation in selected clinical scenarios, they do not consistently support durable melanocyte regeneration or restore immune tolerance including the specific dampening of IFN-γ-dependent inflammatory amplification, IL-15-mediated maintenance of T_RM_ cells, and the persistence of pathogenic memory T-cell populations. As a result, these approaches rarely achieve durable immune tolerance or long-term disease remission.

This recognition has driven a shift toward therapeutic approaches that more directly engage disease specific mechanisms and aim to promote long-term immune regulation together with functional recovery of the melanocyte compartment. Within this evolving therapeutic landscape, ultraviolet (UV) based phototherapy (described in detail latter in this review) occupies a distinctive position. Unlike purely immunosuppressive interventions, phototherapy exerts simultaneous effects on immune modulation and melanocyte activation, thereby representing a transitional strategy that links control of pathogenic immune responses with stimulation of cutaneous regeneration.

### Targeted Immunomodulation and Cytokine-driven Therapies

#### JAK Inhibitors and TYK2 Inhibitors

Targeted immunotherapies based on small molecules and monoclonal antibodies have progressively advanced from preclinical investigation to early and late phase clinical trials in vitiligo. Many of these agents were initially developed for other autoimmune or inflammatory diseases and were subsequently repurposed based on shared pathogenic mechanisms. In vitiligo, this therapeutic transition has been driven by the identification of cytokine-dependent signaling networks that sustain autoreactive T-cell responses within the skin, particularly those centered on IFN-γ and IL-15 signaling axes. Among them, inhibitors of JAK and tyrosine kinase 2 (TYK2) have received particular attention due to their capacity to modulate cytokine driven signaling pathways central to vitiligo pathogenesis [161, 162].

JAK inhibitors directly target these pathogenic circuits by interfering with multiple members of the JAK family (JAK1, JAK2, and/or JAK3), which are critical mediators of cytokine receptor-associated signaling pathways. As a result, they broadly attenuate downstream STAT-dependent transcriptional programs. For instance, binding of IFN-γ to its receptor complex triggers activation of JAK1 and JAK2, leading to phosphorylation of STAT1 and subsequent induction of CXCL9 and CXCL10, which are key chemokines in the recruitment and epidermal localization of CXCR3⁺ autoreactive CD8⁺ T cells, thereby sustaining a feed-forward inflammatory loop within vitiligo lesions [99, 163]. In parallel, TYK2 inhibition may act upstream by modulating CD4⁺ T-cell polarization, indirectly reducing IFN-γ production and downstream chemokine expression. This selective modulation may attenuate pathogenic immune activation while preserving other JAK-dependent physiological pathways, potentially translating into improved safety profiles [164].

This mechanistic rationale is strongly supported by clinical evidence. Ruxolitinib (Opzelura™), a topical JAK1/2 inhibitor, is the first targeted therapy approved for nonsegmental vitiligo (adults and children 12 years of age and older). Treatment significantly improved facial and total body repigmentation in adolescents and adults with non-segmental vitiligo, as measured by validated VASI endpoints [80], in addition to a favorable safety profile, limited to mild local adverse events and no new systemic signs [81]. In parallel, systemic JAK inhibition with oral ritlecitinib (JAK3/TEC inhibitor) induced clinically significant repigmentation, along with reductions in pro-inflammatory biomarkers at the transcriptional and protein levels, which correlated with increased markers associated with melanocytes. Notably, therapeutic response appears to depend on disease activity, with faster repigmentation in stable lesions and delayed responses in active disease requiring prior immunological control [165–167]. Consistently, oral upadacitinib (selective JAK1 inhibitor) also demonstrated significant and sustained improvements in facial and total body repigmentation in a phase II randomized trial [168].

Other agents currently under investigation include topical ivarmacitinib (NCT 04774809), topical cerdulatinib (NCT 04103060), oral baricitinib (NCT 04822584; NCT 05950542), oral povorcitinib (NCT 06113471; NCT 06113445), oral deucravacitinib (NCT 06327321), oral zasocitinib (NCT 07108283), and oral tofacitinib in combination with phototherapy (NCT 07044141).

#### IFN-γ / CXCL10 / CXCR3 Axis

Kinase inhibition strategies primarily attenuate upstream cytokine signaling. Nevertheless, effective disease control in vitiligo also requires direct interference with downstream effector mechanisms responsible for immune cell recruitment and melanocyte damage within the skin microenvironment. As described before, IFN-γ signaling induces CXCL10 expression and promotes CXCR3 dependent trafficking of cytotoxic T cells to the skin, thereby sustaining cutaneous inflammation [99, 163]. Importantly, IFN-γ signaling not only regulates immune cell trafficking but also reinforces effector function within the skin. In murine models of vitiligo, IFN-γ has been shown to be essential for the accumulation of autoreactive CD8⁺ T cells in lesional skin, and its neutralization prevents disease progression and reduces T-cell infiltration [103]. Furthermore, CXCR3 expression on CD8⁺ T cells is critical not only for their recruitment but also for their retention and positioning within the epidermis, where melanocytes reside. Disruption of the CXCL10-CXCR3 axis significantly impairs T-cell trafficking and attenuates depigmentation in experimental models, underscoring its role as a non-redundant pathway in disease maintenance [99, 163].

Systemic IFN-γ blockade is constrained by its critical role in antimicrobial defense and tumor immunosurveillance. To address this limitation, a bispecific antibody was engineered to neutralize IFN-γ while anchoring to keratinocytes via desmoglein binding, thereby restricting its activity to the epidermal compartment. This spatially targeted approach enables localized inhibition of IFN-γ signaling, resulting in decreased production of CXCL9 and CXCL10. In a murine model of vitiligo, this strategy effectively limited CD8⁺ T-cell accumulation in the skin and led to significant disease improvement [169]. While these findings support localized immunomodulation, the extent to which systemic immune functions are preserved remains to be fully determined [169].

Consistent with the central role of this pathway, genetic and pharmacological studies have demonstrated that CXCL10 is a non-redundant mediator of disease progression. Rashighi et al. demonstrated that Cxcl10 deficient mice, as well as mice treated with CXCL10 neutralizing antibodies, exhibited significantly reduced depigmentation and diminished accumulation of autoreactive CD8⁺ T cells in the skin. Notably, CXCL10 blockade in established lesions promoted partial repigmentation, indicating that this chemokine contributes not only to disease initiation but also to its maintenance [163]. Subsequent work from the same group further refined this concept by demonstrating that direct depletion of CXCR3⁺ T cells induces more durable perifollicular repigmentation compared with chemokine neutralization alone. Mechanistically, this approach eliminates autoreactive effector cells rather than solely impairing their migration, thereby disrupting both ongoing tissue damage and the reservoir of pathogenic T cells capable of reinitiating disease [170].

Together, these findings establish the IFN-γ-CXCL10-CXCR3 pathway as a central effector axis linking cytokine signaling to T-cell recruitment, epidermal positioning, and melanocyte destruction in vitiligo. However, the persistence and recurrence of disease activity cannot be fully explained by immune cell recruitment alone. Increasing evidence indicates that long-lived tissue-T_RM_ cells persist within the skin and retain the capacity to rapidly reinitiate cytotoxic responses upon antigen re-exposure, even in the absence of continuous recruitment from the circulation [97].

These observations suggest that effective therapeutic strategies must not only disrupt inflammatory recruitment pathways but also target the mechanisms that sustain local immune memory and pathogenic persistence over time.

#### IL-15 and Tissue T_RM_ Cells

Further studies expanded this concept by identifying IL-15 as a critical regulator of the survival, metabolic fitness, and effector function of epidermal CD8⁺ T_RM_ cells. In vitiligo lesions, keratinocytes express IL-15 receptor alpha (IL-15Rα/CD215), enabling the trans-presentation of IL-15 to neighboring CD122⁺ (IL-2/15Rβ) T_RM_ cells. This process facilitates the formation of a high-affinity receptor complex that activates JAK1 and JAK3, leading to STAT5 phosphorylation and transcriptional programs that promote T-cell survival, proliferation, and sustained effector function [150]. Through this mechanism, T_RM_ cells are maintained as a long-lived, self-sustaining population within the epidermal niche. Functionally, these cells localize to the dermoepidermal junction, where they continuously or intermittently produce IFN-γ, thereby reinforcing local chemokine production and maintaining a pro-inflammatory microenvironment even in the absence of ongoing recruitment from the circulation. This establishes a localized immune memory circuit that contributes to disease persistence and rapid relapse following treatment discontinuation [97]. Consistent with this pathogenic role, experimental blockade of IL-15 signaling has been shown to selectively disrupt T_RM_ cell maintenance. In murine model of vitiligo, antibody-mediated targeting of CD122 resulted in depletion of epidermal CD8⁺ T_RM_ cells, reduced IFN-γ production, and induced durable repigmentation, even after cessation of therapy [108].

Building on these findings, early-phase clinical studies are now evaluating therapeutic strategies targeting the IL-15 axis in patients with vitiligo. A Phase 1b trial is assessing the safety and tolerability of the anti-IL-15 monoclonal antibody TEV-53408 (NCT06625177). In parallel, AMG 714, an anti-IL-15 monoclonal antibody, has been investigated in a Phase 2 clinical study, including in combination with narrowband UVB phototherapy (NCT04338581). Additionally, FB102, an anti-CD122 monoclonal antibody designed to block IL-2 and IL-15 receptor signaling, is currently being evaluated in a randomized, double-blind, placebo-controlled Phase 1 trial in patients with non-segmental vitiligo (NCT06905873).

Altogether, these strategies aim to disrupt a non-redundant survival pathway for pathogenic T_RM_ cells, thereby targeting a key mechanism underlying disease persistence. In contrast to therapies that primarily inhibit immune activation or cellular recruitment, IL-15-directed interventions seek to eliminate or destabilize the local reservoir of autoreactive memory T cells within the skin.

However, within the broader conceptual framework in which vitiligo persistence is sustained by coordinated interactions between tissue-resident and systemic immune memory compartments, targeting T_RM_ cells alone may be insufficient to achieve durable disease control. Current therapies, including JAK inhibitors, primarily suppress cytokine signaling pathways such as IFN-γ-JAK-STAT, effectively reducing inflammation and promoting repigmentation, but do not fully eliminate autoreactive memory T-cell populations that may persist across compartments [80].

Similarly, although IL-15 blockade can disrupt T_RM_ maintenance and induce durable repigmentation in experimental models, these approaches may not fully address circulating or systemic memory populations, which can re-seed the skin and contribute to disease recurrence. This highlights that vitiligo is sustained not only by local immune circuits but by a distributed memory network that prevents a longstanding immunological control of the responses. Therefore, durable disease control in vitiligo will likely require therapeutic strategies capable of targeting both local and systemic immune memory, as well as promoting antigen-specific immune tolerance. This may include combination approaches integrating cytokine blockade, memory-cell modulation, and regenerative therapies aimed at restoring melanocyte homeostasis.

In parallel, complementary therapeutic strategies continue to target broader IFN-driven inflammatory programs that contribute to immune amplification and disease propagation, highlighting the need for integrated approaches that simultaneously address immune activation, memory, and tissue repair.

#### Type I IFN Blockade

Type I IFN, including IFN-α and IFN-β, have emerged as important upstream regulators of innate and adaptive immune activation in vitiligo. These cytokines are primarily produced by pDCs in response to cellular stress and damage-associated molecular patterns released by melanocytes and keratinocytes. Upon binding to the type I IFN receptor (IFNAR1/IFNAR2), IFN-I activates JAK1 and TYK2, leading to phosphorylation of STAT1 and STAT2 and induction of IFN-stimulated genes (ISGs), which amplify inflammatory signaling and promote antigen presentation.

In the context of vitiligo, experimental evidence indicates that activation of pDCs and subsequent IFN-α production enhance antigen presentation and promote the activation and effector differentiation of melanocyte-reactive CD8⁺ T cells. In addition, type I IFN signaling increases MHC class I expression and sensitizes keratinocytes and immune cells to inflammatory stimuli, thereby facilitating the amplification of IFN-γ–dependent cytotoxic responses within the skin [29, 171].

Anifrolumab is a fully human monoclonal antibody that targets the IFNAR1 subunit, thereby blocking signaling induced by all type I IFN. This inhibition prevents activation of the JAK1-TYK2-STAT1/STAT2 pathway and reduces the transcription of IFN-stimulated genes involved in immune activation and inflammation. Clinically, anifrolumab has been approved by the United States Food and Drug Administration and the European Medicines Agency for the treatment of moderate to severe systemic lupus erythematosus, a disease strongly driven by type I IFN signaling [172].

Hence, based on the involvement of type I IFN in vitiligo pathophysiology, a clinical study is currently evaluating the efficacy and tolerability of anifrolumab infusions in combination with NB UVB phototherapy in patients with progressive disease (NCT05917561). The rationale for this approach lies in the potential to interfere with early innate immune activation and antigen presentation, thereby limiting the priming and expansion of autoreactive T cells that subsequently sustain IFN-γ-mediated tissue damage.

Beyond cytokine-directed interventions, additional therapeutic strategies have focused on restoring immune tolerance by modulating inhibitory pathways that regulate pathogenic T-cell activity.

#### Immune Checkpoint Modulation

From a complementary perspective aimed at restraining pathogenic T cell activity through inhibitory immune checkpoints, modulation of the PD-1/PD-L1 axis has emerged as a biologically relevant strategy to restore immune tolerance in vitiligo. In experimental model of vitiligo, enhancement of PD-1 signaling has been shown to suppress pathogenic immune responses. Miao et al. demonstrated that systemic administration of a PD-L1 fusion protein in the Pmel-1 mouse model resulted in a significant reduction in depigmentation, with approximately 58% improvement compared with untreated controls. Mechanistically, this effect was associated with a marked expansion of Tregs in both skin and lymphoid organs, alongside a substantial reduction in effector T cells, indicating a shift toward a more regulated immune state [6]. These findings are consistent with the broader role of PD-1 signaling in promoting T-cell exhaustion and functional restraint, particularly within chronically inflamed tissues. In the skin, engagement of the PD-1/PD-L1 axis may limit the cytotoxic activity of CD8⁺ T cells and modulate the balance between effector and regulatory compartments, thereby counteracting the IFN-γ-driven inflammatory circuits that sustain melanocyte destruction.

Despite this strong preclinical rationale [173–176], there are currently no clinical studies evaluating PD-1/PD-L1 agonist strategies in patients with vitiligo. Moreover, therapeutic enhancement of checkpoint signaling raises important safety considerations. Systemic or even localized immunosuppression may impair host defense against infections and tumor surveillance, particularly in the skin, which serves as a critical barrier tissue. In addition, excessive inhibition of T-cell activity could potentially interfere with normal immune surveillance mechanisms or lead to unintended alterations in tissue-resident immune populations, including regulatory and memory T-cell subsets. Another key limitation lies in the incomplete understanding of how PD-1 modulation affects T_RM_ cells, which are central to disease persistence. While PD-1 signaling may restrain effector function, it is unclear whether it can effectively eliminate or reprogram pathogenic T_RM_ populations, raising the possibility that checkpoint-based therapies may suppress inflammation without achieving durable immune resolution.Taken together, these observations indicate that although immune checkpoint modulation represents a biologically plausible strategy to restore peripheral tolerance in vitiligo, its clinical translation requires careful evaluation of efficacy, specificity, and safety. Future studies should aim to define whether targeted or tissue-restricted approaches can harness the immunoregulatory potential of checkpoint pathways while minimizing systemic immunosuppressive risks.

Together, these observations establish immune checkpoint pathways as critical regulators of immune balance in vitiligo, linking defective inhibitory signaling to sustained autoreactive T-cell activity. While still underexplored therapeutically, strategies aimed at enhancing checkpoint signaling may offer a means to restore immune tolerance and counteract the failure of durable immune control that characterizes the disease.

In parallel with efforts to reestablish immune regulation through cytokine-targeted therapies and checkpoint engagement, additional innovative pharmacological approaches are necessary to fully address the underlying metabolic dysfunction that sustains immune activation and melanocyte vulnerability.

### Immunometabolic Therapeutic Strategies: Targeting Oxidative Stress and Metabolic Reprogramming

#### Selective Immunomodulators With Metabolic Effects

Emerging therapeutic strategies in vitiligo increasingly recognize that immune dysregulation is tightly coupled to metabolic dysfunction. Rather than acting solely on cytokine signaling, these approaches aim to modulate immunometabolic pathways that govern redox balance, mitochondrial integrity, and inflammatory activation, thereby addressing both melanocyte vulnerability and immune persistence.

One of the central targets within this framework is oxidative stress. Pharmacological activation of the Nrf2-ARE pathway has been shown to restore antioxidant defenses and protect melanocytes from oxidative damage. For instance, Ma J. et al. demonstrated that baicalein enhances Nrf2 signaling, reduces intracellular reactive oxygen species, and prevents apoptosis in human vitiligo melanocytes [177]. However, these findings are currently limited to in vitro studies and preclinical models, with no clinical trials in patients with vitiligo to date. Similarly, Chang Y. et al. showed that simvastatin improves cellular resistance to oxidative stress [178]. However, although simvastatin is clinically approved for other indications, its use in vitiligo remains experimental and has not been validated in controlled clinical trials.

In addition to redox modulation, targeting mitochondrial dysfunction has emerged as a promising strategy. Recent studies have shown that inhibition of VDAC1 oligomerization reduces mitochondrial DNA release and attenuates activation of the cGAS–STING pathway, thereby limiting innate immune activation and inflammatory amplification in vitiligo [179]. These approaches remain at the preclinical stage and have not yet been translated into human studies.

Another important metabolic target is the inflammasome pathway. Tranilast, an inhibitor of NLRP3 inflammasome activation, has been shown to suppress IL-1β production and prevent inflammation-induced melanocyte dysfunction [38]. Importantly, this mechanistic effect has been described in preclinical and in vitro models, and there are currently no clinical trials evaluating tranilast specifically for vitiligo.

Ferroptosis has also been proposed as a novel therapeutic target. Alterations in iron metabolism and lipid peroxidation pathways contribute to melanocyte death in vitiligo, suggesting that ferroptosis inhibitors may provide cytoprotective effects and preserve the melanocyte pool [43]. However, therapeutic targeting of ferroptosis in vitiligo remains an emerging concept without clinical validation.

Within this immunometabolic framework, previously described agents can be reinterpreted as modulators of metabolic-immune interactions rather than solely immunosuppressive drugs. Lenalidomide, an immunomodulatory derivative of thalidomide, exemplifies this dual activity by modulating cytokine production and immune cell function. In a murine model of vitiligo, oral administration of lenalidomide for six weeks significantly reduced lesion development and suppressed key pro-inflammatory cytokines, including IFN-γ, tumor necrosis factor (TNF), and IL-6, while concomitantly increasing anti-inflammatory mediators such as IL-4 and IL-10. Notably, these effects occurred without significant changes in the CD4⁺/CD8⁺ T-cell ratio, suggesting functional reprogramming rather than depletion of immune subsets [180]. Despite these promising results, lenalidomide has not been evaluated in clinical trials for vitiligo and remains a preclinical or experimental candidate.

Another relevant class of immunometabolic modulators comprises phosphodiesterase 4 (PDE4) inhibitors, which exert anti-inflammatory effects by increasing intracellular cyclic adenosine monophosphate (cAMP) levels. Elevated cAMP activates protein kinase A (PKA), which can suppress pro-inflammatory signaling pathways, including NF-κB, leading to reduced cytokine production [181]. In melanocytes, cAMP signaling enhances MITF activity and promotes melanogenesis [182]. Although PDE4 inhibition may theoretically support melanocyte function through cAMP elevation, direct evidence demonstrating that specific PDE4 inhibitors enhance melanocyte proliferation and melanogenesis remains limited and primarily restricted to preclinical models and to a 3-dimensional skin equivalent model [183, 184]. Clinically, PDE4 inhibitors have shown efficacy in inflammatory skin diseases [185]; however, their role in pigmentary disorders such as vitiligo remains investigational, with limited clinical evidence [186]. Although promising, these results still require validation in larger trials.

Beyond these approaches, emerging nanotechnology-based antioxidant therapies have demonstrated the capacity to simultaneously modulate redox balance and immune activation [187], showed that targeted delivery of antioxidant nanoparticles reduces ROS levels, suppresses CD8⁺ T-cell infiltration, and promotes repigmentation in experimental models, highlighting the potential of combined immunometabolic interventions. These approaches remain strictly preclinical and have not yet entered clinical testing.

Overall, most immunometabolic interventions in vitiligo are currently restricted to preclinical or early-phase clinical studies, with only PDE4 inhibitors showing preliminary translational potential in non-segmental vitiligo. Collectively, these findings support a model in which targeting metabolic dysfunction may simultaneously protect melanocytes and attenuate immune activation. By addressing oxidative stress, mitochondrial signaling, inflammasome activation, and metabolic cell death pathways, immunometabolic therapies offer a complementary strategy to conventional immunosuppression.

Importantly, such approaches may also contribute to restoring immune resolution by reducing chronic inflammatory signaling and destabilizing pathogenic tissue-resident memory T-cell populations. However, their clinical applicability remains to be established, and careful evaluation of safety, efficacy, and durability of response is required before routine use in clinical practice.

### Other Supportive and Emerging Modalities

#### UV Based Phototherapy as a Bridge Between Immune Modulation and Regeneration

UV-based phototherapy (including psoralen plus UVA (PUVA), UVB, and UVA1) is one of the most established dermatological treatment modalities. In vitiligo, the choice of phototherapy, whether localized or whole-body, is based on the extent, viability, and distribution of the lesion [188]. The suppression of the immune response is mediated by several neuroimmunoendocrine mechanisms, including the isomerization of urocanic acid, vitamin D formation, altered functioning of antigen-presenting cells and production of soluble mediators [189–191]. Regarding its pro-melanogenic effects, stimulation is also mediated by several mechanisms, including indirectly by increasing the production of L-DOPA, a precursor of melanin, and catecholamines; increased p53 and proopiomelanocortin (POMC)-derived peptides (e.g. ACTH, α-MSH, β-endorphin) expression and other melanocyte growth factors by keratinocytes lead to signaling mediated by MC1R, endothelin receptors, and Wnt receptors in melanocytic stem cells [192–198].

In current clinical practice, narrowband UVB (NB-UVB) therapy shows higher response rates compared to PUVA, with a shorter treatment period, and improved patient safety [130, 131, 199]. Therefore, doses of 200 mJ/cm² (light skin) to 400–500 mJ/cm² (darker skin) are indicated according to skin color and lesion location, with up to three sessions per week, achieving repigmentation rates above 75% within 12 months [144, 199, 200]. In addition, phototherapy combined with immunosuppressants [168, 201] and JAK inhibitors [169–171, 202] is also a strategy with better patient adherence and improved repigmentation rates. For localized and sensitive lesions, such as those on the face, another alternative has been the 308 nm monochromatic laser and excimer lamp [132, 133, 164], with similar or even greater efficacy than NB-UVB [203, 204]. Synergistically, the combination with JAK inhibitor and 308 nm excimer laser demonstrated significant effects on repigmentation, mainly in facial lesions, accompanied by low recurrence rates after one year (8.8%) [78].

Despite these advantages, patient dissatisfaction with available treatments remains high, with more than 90% of individuals with vitiligo reporting the need for more effective therapeutic options [205]. This highlights the persistent challenge of achieving durable immune resolution and sustained repigmentation, even with modalities that partially address both arms of disease pathophysiology.

Taken together, these limitations highlight the need for rationally engineered and combination based therapeutic strategies capable of simultaneously integrating immune modulation, resolution of chronic inflammation, and tissue regeneration. In this context, advances in therapeutic engineering and precision immunoengineering are increasingly recognized as essential for improving vitiligo management, with the goal of limiting disease progression and recurrence while preventing irreversible melanocyte loss.

A direct consequence of this shift has been the development of therapies designed to selectively target key cytokine driven pathways that sustain immune activation and melanocyte damage in vitiligo. By intervening at defined molecular nodes within pathogenic immune circuits, these approaches aim to move beyond broad immunosuppression toward mechanism-based modulation of disease relevant signaling networks.

#### Promising Therapies Promoting Melanocyte Regeneration and Functional Repigmentation

Beyond immunological control, durable repigmentation in vitiligo critically depends on the restoration of the melanocyte compartment through coordinated processes of proliferation, migration, and differentiation. Importantly, accumulating evidence indicates that this regenerative response is largely driven by melanocyte stem cells (McSCs), which reside primarily within the hair follicle bulge. In humans, additional melanocyte precursor populations have also been identified in the interfollicular epidermis and adnexal structures, suggesting the presence of supplementary reservoirs contributing to repigmentation [188, 189, 206].

McSCs originate from neural crest–derived melanoblasts and persist as a quiescent population within specialized niches that tightly regulate their activation, self-renewal, and differentiation. Upon appropriate stimulation, including ultraviolet radiation or pharmacological signals, McSCs exit quiescence, migrate along the outer root sheath, and differentiate into mature melanocytes that repopulate depigmented epidermis, giving rise to the characteristic perifollicular pattern of repigmentation observed in vitiligo [190, 191].

This process is tightly regulated by niche-derived signaling pathways, including Wnt/β-catenin, Notch, TGF-β, and KIT signaling, which collectively maintain McSC quiescence or promote activation and lineage commitment depending on the microenvironmental context [192, 193]. Disruption of these regulatory circuits has been implicated in impaired melanocyte regeneration and disease persistence in vitiligo.

Within this regenerative framework, UV phototherapy remains a cornerstone of treatment, not only due to its immunomodulatory effects but also because of its capacity to activate McSCs and promote their migration into the epidermis. UV-induced signaling enhances KIT ligand expression, stimulates melanocyte proliferation, and facilitates differentiation through activation of MITF-dependent transcriptional programs [194–196].

In parallel, pharmacological strategies have been developed to directly enhance melanocyte function and support repigmentation. Prostaglandin (PG) analogues such as latanoprost and bimatoprost, are clinically associated with cutaneous hyperpigmentation and hypertrichosis [197]. Mechanistic studies have further shown that PGE2 signaling through EP receptors activates the cAMP and protein kinase A cascade, enhancing melanocyte dendricity and TYR activity [198, 199]. Consistent with these observations, topical PG analogues have demonstrated significantly higher repigmentation rates than placebo in small clinical studies. In selected localized lesions, clinical responses have approached those achieved with established modalities such as NB-UVB phototherapy [200, 201]. Moreover, both latanoprost and bimatoprost have shown additive benefits when used in combination with narrowband UVB, resulting in improved repigmentation compared with phototherapy alone [202–204].

Other molecules, such as α-MSH analogues, have emerged as a pharmacological option capable of directly activating the melanogenic cascade via the MC1R and activating the cAMP-PKA-CREB-MITF pathway [207, 208]. Randomized and prospective clinical studies have shown that monthly subcutaneous implantation of afamelanotide, a synthetic α-MSH analog, used in combination with NB-UVB phototherapy, significantly enhances repigmentation scores and accelerates treatment responses, with particularly pronounced effects in facial and truncal regions [209, 210]. A clinical trial is currently evaluating the efficacy of a sustained release afamelanotide implant administered every three weeks in combination with NB-UVB in patients with stable or active generalized disease (NCT06109649). Preclinical studies also suggest that microneedle based delivery of α-MSH increases dermal bioavailability and augments melanogenic activity, supporting minimally invasive delivery platforms as promising tools for pro-melanogenic therapies [211].

Efforts to develop more selective MC1R agonists have led to the identification of stable and potent melanocortin analogues, including small-molecule and peptidomimetic compounds, capable of inducing melanogenesis independent of UV exposure. Preclinical studies have shown that selective MC1R activation enhances UV-induced DNA repair responses and reduces markers of genotoxic stress in melanocytes [212]. In this context, a self-assembling melanocortin peptide agonist capable of forming a hydrogel after subcutaneous administration has shown marked increases in the expression of TYR and related melanogenic enzymes, resulting in a several fold increase in melanin content in preclinical models [213].

Complementary to melanocortin signaling, ion channel-mediated pathways have recently emerged as additional regulators of melanogenesis. The transient receptor potential ankyrin 1 (TRPA1) channel, a calcium-permeable sensor activated by UVB radiation, has been implicated in the control of skin pigmentation. TRPA1 activation induces intracellular Ca²⁺ influx, regulates melanosomal luminal pH, and enhances TYR activity. In pigmented guinea pigs, topical pretreatment with the TRPA1 agonist JT010 significantly augmented UVB-induced pigmentation, with increased melanin deposition and upregulation of TYR and TRPA1 expression in basal melanocytes. Together, these findings suggest that pharmacological activation of TRPA1 may represent a potential adjunct strategy to enhance the efficiency of UV-based repigmentation therapies [214].

Growth factors such as basic fibroblast growth factor (bFGF) have emerged as potential adjuvants in melanocyte regulation. Experimental studies have shown that NB-UVB exposure promotes melanocyte proliferation and migration, effects that are thought to be mediated, at least in part, by keratinocyte-derived growth factors and activation of intracellular survival pathways [215, 216]. Additional signaling, including extracellular signal-regulated kinase (ERK) and c-Jun N-terminal kinase (JNK) activation and increased expression of phosphorylated focal adhesion kinase, have been implicated in bFGF-mediated melanocyte migration [217]. Insulin like growth factor 1 also exerts protective effects by reducing expression of inflammatory mediators, limiting CD8^+^ T cell infiltration, and restoring antioxidant responses in keratinocytes, thereby mitigating oxidative stress induced depigmentation [218]. Taken together, this support framework based on growth factor signaling provides a mechanistic foundation for broader regulatory and regenerative circuits that integrate local cutaneous signaling in the control of repigmentation.

#### Neuroendocrine and Stress-responsive Modulation of Pigmentation

Recent studies have uncovered emerging regulatory axes involving neurotransmitters and hormonal signaling in melanogenesis. However, increasing evidence indicates that these neuroendocrine mediators also play a central role in shaping immune responses in vitiligo, establishing a bidirectional neuro-immune-cutaneous axis that contributes to both melanocyte dysfunction and autoimmune activation.

Tang et al. demonstrated that serotonin (5-HT) and its receptor 5-HT7 function as pro-melanogenic factors, with expression levels of 5-HT7, TYR, and MITF significantly reduced under stress conditions but restored following transdermal treatment with LP-12, a potent 5-HT7 receptor agonist. LP-12 promoted melanin synthesis, dendrite elongation, and chemotactic motility of melanocytes both in vitro and in vivo, effects mediated through activation of cAMP-PKA-ERK1/2, classical mitogen-activated protein kinase (MAPK), RhoA/Rab27a, and phosphoinositide 3-kinase (PI3K)/protein kinase B (AKT) signaling pathways [219].

Supporting the role of the local serotonergic system in pigment homeostasis, studies in C57BL/6 mouse skin show that stress impairs cutaneous 5-HT signaling and is associated with reduced melanogenic activity, including decreased expression of melanogenesis-related proteins [220]. Pharmacological interventions such as 5-hydroxy-L-tryptophan, fluoxetine, or 5-HT1A/1B receptor agonists attenuate stress-induced hypopigmentation and restore melanogenic function. Notably, fluoxetine-mediated pigmentation improvement involves activation of p38 MAPK signaling and upregulation of Rab27a, a key GTPase involved in melanosome transport, through differential engagement of 5-HT receptor subtypes [220, 221]. These findings highligh the role of serotonergic signaling in both melanocyte function and intracellular pigment trafficking and further suggest that stress-induced disruption of serotonergic signaling may not only impair melanocyte function but also contribute to a permissive environment for immune-mediated melanocyte damage.

Importantly, neuroimmune crosstalk further modulates this pathway. IFN-γ, a central cytokine in vitiligo pathogenesis, suppresses 5-HT-induced melanogenesis by downregulating 5-HT receptors, demonstrating a direct inhibitory effect of immune activation on neuroendocrine melanogenic signaling [222]. This establishes a feedback loop in which inflammatory cytokines impair neuroendocrine pigmentation pathways.

Beyond serotonergic signaling, activation of the hypothalamic-pituitary-adrenal (HPA) axis represents a key interface between stress and immune dysregulation. Under these conditions, substance P (SP) suppresses melanogenesis indirectly through keratinocyte-mediated signaling involving the HPA axis, resulting in reduced melanogenic activity and downregulation of melanogenesis-related proteins, including TYR. These findings provide mechanistic evidence that stress-responsive neuropeptides can modulate pigmentation through endocrine-like signaling within the skin microenvironment [223]. On the other hand, neuropeptides derived from cutaneous nerve fibers further amplify immune responses in vitiligo. Calcitonin gene-related peptide (CGRP), released from nociceptive neurons, enhances cDC1 activation and promotes autoreactive CD8⁺ T cell responses, directly linking neuronal signaling to melanocyte destruction [224]. In addition, dysregulated CGRP signaling impairs Treg expansion under oxidative stress conditions, further exacerbating immune imbalance [225].

Complementing these mechanisms, neuropeptide Y represents another critical mediator of neuroimmune communication. Laddha et al. demonstrated that genetic variants associated with increased NPY expression correlate with elevated IL-1β levels and increased susceptibility to vitiligo [226]. Mechanistically, NPY acts on multiple immune cell populations, including T cells, B cells, dendritic cells, and macrophages, promoting cytokine production and inflammatory responses. These findings reinforce the concept that neuropeptides can directly modulate immune pathways involved in melanocyte destruction. Consistent with these observations, activation of the sympathetic nervous system has emerged as a key driver of neuroimmune dysregulation in vitiligo. Norepinephrine released from sympathetic nerve endings promotes CXCL9 and CXCL10 production, enhancing recruitment and activation of CXCR3⁺ CD8⁺ T cells and amplifying IFN-γ-dependent inflammation [227].

Complementing these findings, estrogen signaling has been implicated in melanocyte protection against oxidative stress. Yamamoto et al. demonstrated that treatment with 17β-estradiol significantly attenuates oxidative damage in primary human epidermal melanocytes exposed to hydrogen peroxide. The authors further showed that oxidative stress conditions associated with vitiligo reduce 17β-hydroxysteroid dehydrogenase type 1 expression in keratinocytes, resulting in decreased local estradiol production and compromised melanocyte resistance to oxidative injury [228].

From a translational perspective, targeting neuroendocrine pathways has shown clinical promise. Lim et al. demonstrated that pharmacological activation of the melanocortin system using the α-MSH analogue afamelanotide significantly enhances and accelerates repigmentation when combined with NB-UVB phototherapy [209]. This highlights the therapeutic potential of restoring neuroendocrine signaling to promote melanocyte regeneration.

Collectively, these findings support a model in which neuroendocrine mediators not only regulate melanocyte biology but also actively shape the cutaneous immune microenvironment. Stress-induced neuroendocrine activation promotes inflammatory signaling, enhances antigen presentation, and drives autoreactive CD8⁺ T cell responses, while inflammatory cytokines reciprocally disrupt melanogenic and neuroendocrine pathways. This bidirectional crosstalk establishes a self-amplifying loop that sustains disease activity. Therefore, integrating neuroendocrine mediators into therapeutic design could enable alternative next-generation approaches for vitiligo treatment. In this context, several emerging interventions such as cellular modulation have attracted attention for their potential to restore homeostasis and support more durable repigmentation outcomes. Beyond modulating immune and metabolic pathways, durable repigmentation ultimately requires restoration of the melanocyte compartment and its regenerative niche.

### Regenerative and Cell-based Therapies: Restoring the Melanocyte Niche

#### Cellular and Tolerogenic Therapeutic Approaches

Cell therapy, defined as the administration of living cells as therapeutic agents, has emerged as a highly advanced strategy within modern biomedical and regenerative medicine. Its promise lies in the ability to engineer or redirect cellular functions, enabling therapeutic modalities with unique mechanisms of action and the potential for improved specificity and durability compared with conventional pharmacological approaches [229].

In vitiligo, the most established cellular approaches involve tissue grafts and cell suspension techniques, which are primarily indicated for segmental vitiligo and stable non-segmental disease that is refractory to medical treatment. Traditional tissue grafting methods, including split thickness skin grafts, suction blister grafts, and punch grafts, have been used clinically for several decades. More recently, cellular grafting techniques have undergone substantial refinement and standardization. These approaches typically involve separation of the epidermis and dermis to generate epidermal cell suspensions that may be applied directly or after in vitro expansion. Cultured melanocytes, non-cultured epidermal cells, and hair follicle derived cell suspensions represent additional therapeutic options within this category [230].

In a recent clinical study, transplantation of follicle-derived melanocyte lineage cells resulted in marked repigmentation in a high proportion of treated lesions, reaching approximately 92% in this preliminary cohort [231]. Furthermore, a recent meta analysis demonstrated that the highest rates of near complete repigmentation, defined as 90% or greater, were achieved with split thickness skin grafts, followed by suction blister grafts, cultured epidermal cell suspensions, non-cultured epidermal cell suspensions, punch grafts, and non-cultured follicular cell suspensions. Importantly, treatment outcomes varied according to patient age, lesion distribution, anatomical location, and disease stability, underscoring the need for careful patient selection and individualized therapeutic planning [230]. Repigmentation outcomes may be further improved through combined epidermal and follicular cell suspension techniques, which have been associated with superior clinical responses compared with epidermal cell suspensions alone in patients with stable vitiligo [86, 88]. Notably, the success of melanocyte-keratinocyte transplantation is strongly influenced by the local immune microenvironment. Increased lesional CD8^+^ T cell density and elevated CXCL9 expression have been consistently associated with poorer surgical outcomes, highlighting the importance of immune modulation in conjunction with cellular replacement strategies [87, 232]. Accordingly, adjunctive immunomodulatory and phototherapeutic interventions have been explored to enhance the efficacy of cell transplantation. The use of JAK inhibitors, narrowband ultraviolet B phototherapy, and excimer light-based treatments has been shown to accelerate and stabilize repigmentation when combined with cellular therapies in selected clinical contexts [89–91, 233].

Beyond direct melanocyte replacement, mesenchymal stromal cells (MSC) have gained increasing attention as a tolerogenic and supportive cellular therapy. These cells exert their therapeutic effects predominantly through paracrine signaling and immune regulation rather than direct differentiation into melanocytes. MSC are readily isolated, display self renewal capacity, and exhibit broad immunomodulatory properties [234].

In the skin, dermal MSC suppress the proliferation and effector function of skin homing CD8^+^ T cells, thereby creating a more permissive immune environment for melanocyte survival and transplantation [235]. In addition, MSC and their secreted factors support melanocyte survival by downregulating phosphatase and TENsin homolog (PTEN), activating the PI3K Akt pathway, and modulating Wnt beta catenin signaling [236, 237]. Experimental studies have further shown that the combination of MSC with capsaicin reduces HSP70 expression, limits melanocyte apoptosis, improves mitochondrial function, and enhances the expression of melanocytic markers through inhibition of the HSP70/TLR4/mTOR/FAK signaling axis [238].

Induced pluripotent stem cells (iPSCs) represent an additional and highly promising experimental source for cellular therapies in vitiligo. Given the limited availability and proliferative capacity of primary melanocytes, the generation of melanocytes from iPSCs offers a renewable alternative to other cellular sources, including melanocytic stem cells and hair follicle stem cells. Under defined differentiation conditions, iPSCs give rise to melanocytes expressing key lineage markers such as MITF, TYR, and TYRP1, and produce melanosomes that closely resemble those of primary melanocytes in both structure and function. These induced melanocytes are capable of integrating into reconstructed epidermal equivalents in vitro, supporting their potential applicability in regenerative strategies for vitiligo [239–241]. Using a modified in vivo hair follicle reconstitution model, patient derived iPSCs melanocytes localized to the hair bulb and epidermis of recipient mice and produced melanin, predominantly within the hair follicle and epidermis. Melanin production was sustained for up to seven weeks following transplantation, corresponding to a complete hair cycle in this model. These findings demonstrate the feasibility of generating patient-specific melanocytic cell sources and provide experimental insights into melanocyte maintenance, relevant to vitiligo pathophysiology [242].

Innovative cellular reprogramming strategies further expand the therapeutic landscape. Functional melanocytes have been generated from fibroblasts through direct lineage reprogramming. The combined expression of MITF, SOX10, and PAX3 was sufficient to convert murine and human fibroblasts into mature melanocytes capable of producing melanin and integrating into the dermo epidermal junction [243]. Similarly, keratinocyte-lineage cells can be reprogrammed into melanocytes using distinct transcription factor combinations, including MITF, PAX3, SOX2, and SOX9 [244]. Alternative approaches, such as modulation of TAF4 splicing, have also been proposed to activate melanocytic differentiation programs in human dermal fibroblasts, although further studies are required to establish the stability and completeness of this process [245]. In addition, multilineage differentiating stress enduring (Muse) cells isolated from human dermal fibroblasts have been shown to differentiate into functional melanocytes expressing canonical melanocytic markers and producing substantial amounts of melanin. Melanogenesis was maintained following transplantation of engineered skin equivalents into immunodeficient mice, supporting the functional stability of these cells in vivo [246, 247]. Muse exhibit self renewal capacity and can be efficiently isolated from fibroblasts of scalp, further supporting their translational potential [248].

Together, these advances illustrate the increasing sophistication of cellular and tolerogenic therapeutic strategies for vitiligo. By integrating cell replacement, immune modulation, and regenerative engineering, these approaches represent a critical component of next generation precision therapies aimed at restoring durable pigmentation and immune homeostasis in vitiligo.

### Extracellular Vesicles and Intercellular Communication-based Therapies

Building on the advances achieved with cellular and tolerogenic therapies, increasing attention has been directed toward extracellular vesicles as a cell free therapeutic platform capable of recapitulating many of the beneficial effects of parent cells. Extracellular vesicles are nanoscale particles actively generated and secreted by a wide range of cell types into the extracellular environment, where they participate in the regulation of both physiological and pathological processes. Among these vesicles, exosomes, typically ranging from 50 to 150 nm in diameter, transport bioactive cargos including RNA species, proteins, lipids, and in selected contexts DNA, thereby acting as potent mediators of intercellular communication [249, 250].

In the skin, accumulating evidence indicates that extracellular vesicles play an important role in the regulation of melanocyte biology and pigmentation. Vesicles released by melanocytes and enriched in fibronectin have been shown to enhance cell survival following ultraviolet B exposure [251], whereas keratinocyte-derived exosomes regulate melanin synthesis in melanocytes [252]. In parallel, keratinocyte-derived exosomes regulate melanin synthesis in melanocytes and display compositional variability according to skin phototype. Distinct microRNA profiles within these vesicles modulate pigmentation through both MITF dependent and independent mechanisms, including pathways of miR 3196 and miR 203 [252]. Additional studies have demonstrated that keratinocyte derived exosomes enriched in miR 365b 5p enhance melanogenesis by inhibiting GLI2, leading to increased MITF expression and subsequent upregulation of TYR and related melanogenic enzymes [253].

Beyond their effects on pigment production, extracellular vesicles exert broad immunomodulatory functions that are highly relevant to vitiligo pathogenesis. Exosomes derived from MSC have been shown to modulate peripheral blood mononuclear cells by promoting a shift toward Th2 responses, suppressing proinflammatory cytokines such as TNF and IL 1β, and increasing levels of TGF-β and Tregs [254]. In line with these observations, Wang and colleagues investigated the immunoregulatory effects of exosomes derived from three dimensional spheroids of human umbilical cord MSC. Systemic administration of these vesicles ameliorated depigmentation in experimental vitiligo, accompanied by reduced infiltration of CD8 T cells and expansion of Tregs in the skin. Mechanistically, exosomal miR 125b 5p was implicated in reducing oxidative damage in melanocytes, while miR 132 3p contributed to the promotion of Tregs differentiation [255].

Additional studies further support the cytoprotective role of the treatment with MSC exossomes in vitiligo, by showing enhanced melanocyte survival under oxidative stress induced by hydrogen peroxide, in vitro, by downregulating p53, p21, IL 1 beta, IL 6, and IL 8, while restoring TYR activity and increasing MITF and TYRP1 expression [256]. Similarly, exosomes derived from bone marrow MSC attenuated oxidative stress both in vitro and in vivo through hydroxyacyl CoA dehydrogenase-mediated activation of the Nrf2 and HO1 signaling pathway [257].

Encouragingly, these preclinical findings are now beginning to translate into early stage clinical evaluation. Two phase I clinical studies are currently underway to assess the safety and tolerability of exosome-based therapies in patients with localized stable vitiligo (NCT06810869 and NCT06813027). Altogether, these data position extracellular vesicles as a promising therapeutic modality for vitiligo, capable of simultaneously modulating immune responses, protecting melanocytes from oxidative damage, and supporting pigmentation. By harnessing endogenous mechanisms of intercellular communication, the therapies centered on vesicles offer a complementary and potentially transformative extension of cellular approaches within the emerging framework of precision immunoengineering.

### Therapeutic Engineering Platforms Enabling Precision Immunomodulation

#### Therapeutic Nucleic Acids

Genetic engineering approaches are promising strategies to modulate key pathogenic pathways in vitiligo. These therapeutic modalities include RNA-based regulatory tools and DNA-based gene delivery systems, all of which aim to restore immune balance and tissue homeostasis in the context of melanocyte loss [258–260].

Approaches based on RNA have been extensively explored to selectively modulate inflammatory pathways, oxidative stress, and cytokine-driven signaling implicated in vitiligo pathogenesis. In human melanocytes, silencing of HMGB1 using small interfering RNA attenuated oxidative damage and apoptosis induced by hydrogen peroxide, at least in part through activation of the nuclear factor erythroid 2 related factor 2 signaling pathway and upregulation of antioxidant gene expression [261]. Similarly, targeting immune regulatory pathways using RNA interference has shown therapeutic potential. Silencing of interleukin-15 receptor alpha (IL-15Rα) in keratinocytes reduced IL-15 and IL-15Rα expression, impaired IL-15 trans-presentation, and attenuated CD8⁺ T-cell activation under oxidative stress conditions. These findings indicate that RNA-based modulation of the IL-15/JAK/STAT axis may represent an effective strategy to constrain pathogenic immune responses in vitiligo [150].

Further extending this approach, siRNA targeting JAK1 achieved selective gene silencing with minimal effects on other JAK family members. In vitro, JAK1 knockdown suppressed IFN-γ-induced chemokines, including CXCL9, CXCL10, and CXCL11, and demonstrated potent inhibition of inflammatory signaling, in some experimental settings exceeding that observed with the JAK1/2 inhibitor ruxolitinib [262]. In vivo, a single administration resulted in sustained JAK1 silencing for several weeks and significantly reduced depigmentation in a murine model of vitiligo. In human skin explants, JAK1 siRNA was efficiently internalized by fibroblasts, melanocytes, and immune cells, leading to marked reduction in JAK1 transcript levels and attenuation of chemokine release [262]. In parallel, the same group developed siRNA targeting the IFN-γ receptor subunit IFNGR1. Local administration of this construct resulted in sustained receptor knockdown and prolonged reduction of CXCL9 and CXCL10 expression. Broad distribution across epidermal and dermal compartments was observed, with uptake by melanocytes, keratinocytes, fibroblasts, and immune cells [263].

In addition to the prominent results based on RNA products, gene delivery strategies have been investigated to promote immune tolerance and counteract inflammatory amplification loops in vitiligo. Biolistic delivery of a DNA plasmid encoding the HSP70i_Q435A_ mutant promoted repigmentation of skin lesions in the Sinclair swine model, providing evidence for the therapeutic potential of immunomodulatory gene delivery in a translational setting [264]. DNA-based approaches have also been employed to restore defective regulatory immune mechanisms. Impaired recruitment of regulatory T cells to lesional skin has been associated with reduced expression of the chemokine CCL22, which serves as a ligand for the CCR4 receptor expressed on T CD4⁺ and Treg cells [134]. In a murine model of vitiligo, delivery of DNA plasmids encoding Ccl22 led to sustained chemokine expression, enhanced Treg recruitment to the skin, and suppression of cytotoxic anti-melanocyte immune responses, resulting in attenuation of disease progression [265]. Notably, these therapeutic effects were achieved with prolonged activity following a single administration, highlighting the durability of gene delivery interventions.

Collectively, these studies highlight the versatility and precision of RNA and DNA as advanced therapeutic platforms for targeting multiple pathogenic pathways involved in vitiligo autoimmunity. Nevertheless, current reliance on intradermal or localized administration methods limits larger areas application. This limitation underscores the need for alternative delivery strategies, including nanostructured carriers or topical systems, to improve clinical feasibility. Hence, continued advances in delivery technologies are essential for translating nucleic acid-based therapies and other approaches into effective and scalable treatment options for vitiligo.

#### Nanoparticles and Nanotechnology Perspectives

As discussed, in vitiligo and other dermatological diseases, achieving satisfactory therapeutic effects requires that topically applied drugs penetrate the stratum corneum and reach melanocytes and immune cells located in the viable epidermis and dermis. Efficient passive diffusion, whether through appendageal or transepidermal routes, depends on the physicochemical properties of the active molecule, including its shape, molecular weight, pKa, log P, and hydrophilic-lipophilic balance, which determine its transport across the skin barrier [266, 267]. However, only a limited number of compounds possess physicochemical properties compatible with efficient skin penetration. To overcome these constraints, delivery systems based on nanoparticles have emerged as versatile and technologically advanced approaches in bioengineering and pharmacotechnology. These platforms enable modulation of skin penetration pathways, protection of labile molecules, and, in selected contexts, enhanced cellular targeting [258, 268, 269].

Although nanotechnology has been extensively investigated in other dermatological conditions, such as cancer and psoriasis, its application in vitiligo remains relatively limited. Nevertheless, early advances have been reported, particularly using lipid-based nanocarriers. One of the earliest clinical reports was published by De Leeuw et al., who evaluated phosphatidylcholine liposomes stabilized with L-phenylalanine and loaded with khellin, administered in combination with ultraviolet light therapy. Khellin, a naturally occurring furanochromone, has been investigated as a photosensitizing agent for vitiligo treatment when combined with UVA exposure, showing repigmentation effects in small clinical studies [270]. In patients with vitiligo, topical treatment with 0.005% khellin-loaded liposomes combined with ultraviolet radiation was associated with substantial repigmentation after long-term follow-up (approximately 12 months). In a small, non-randomized clinical study, a high proportion of patients achieved marked repigmentation (75–100%) in facial and truncal areas, whereas acral regions such as the hands showed poor response. Based on observational comparisons, repigmentation appeared more pronounced with khellin liposomes combined with UV exposure than with ultraviolet therapy alone [270]. In a subsequent study, the same group reported that the use of khellin-loaded liposomes in combination with ultraviolet radiation following blister roof transplantation resulted in repigmentation exceeding 75% in patients with recalcitrant vitiligo lesions [271].

Beyond the delivery of photosensitizers, lipid nanocarriers have been engineered to improve cutaneous penetration and stability of therapeutic agents. Ultradeformable lipid vesicles (nanospanlastics) loaded with psoralen-rich bergamot oil significantly enhanced skin penetration, vesicle flexibility, and photostability. When used in combination with UVB-based phototherapy over several months, this approach induced variable degrees of repigmentation across different anatomical sites in patients with vitiligo [272].

Similarly, transethosomal nanocarriers loaded with 8-methoxypsoralen enhanced the effects of NB-UVB phototherapy in patients with acral vitiligo, with no significant adverse effects reported during a three-month treatment period [273]. Other examples include ultra-deformable liposomes co-loaded with resveratrol and psoralen, which have been shown in vitro to improve cutaneous delivery and cellular availability of these agents, as well as microemulsions containing *Brosimum gaudichaudii* extracts that directly stimulate melanocyte migration and melanogenesis in vitro [274, 275].

Nanocarriers have expanded the feasibility of topically administering macromolecules, such as nucleic acids, that are otherwise incompatible with passive cutaneous diffusion. In this context, Manosroi et al. developed cationic elastic niosomes to enhance the transdermal delivery, stability, and expression of pMEL34, a TYR-encoding plasmid, highlighting their potential application in vitiligo therapy [276]. These elastic cationic niosomes significantly enhanced tyrosinase expression and activity in melanocytic cell models compared with free plasmid or non-elastic niosomes, and were also shown to increase melanin production in subsequent in vitro studies [276, 277]. Incorporation of the cell-penetrating peptide TAT further enhanced cellular uptake, leading to an order-of-magnitude increase in TYR activity and melanin production in vitro [278].

Expanding these nucleic acid-based strategies targeting melanogenic regulation, our research group demonstrated the potential of liquid-crystalline nanoparticles for topical delivery of RNA molecules [279, 280]. Targeting TYRP1 expression, these nanoparticles efficiently delivered TYRP1-specific siRNA, achieving approximately 80% knockdown of TYRP1 levels within 24 h compared with naked siRNA, in vitro [281].

While previous approaches focus on restoring melanocyte function and regulating melanogenesis, nanotechnology has also enabled strategies targeting the autoimmune component of vitiligo. In this context, Zhang et al. developed polymeric nanoparticles co-encapsulating rapamycin and the melanocyte autoantigen peptide HEL_46−61_, inducing antigen-specific immunological tolerance and preventing vitiligo progression in a murine model [5]. In vitro, dendritic cells efficiently internalized these nanoparticles, resulting in reduced expression of the co-stimulatory molecules CD80 and CD86, suppression of antigen-specific CD4⁺ T-cell proliferation, and promotion of Treg differentiation. In a murine model of vitiligo, repeated intravenous administration attenuated depigmentation, increased local and systemic Treg frequencies, elevated IL-10 levels, and reduced IFN-γ and IL-6, effects that were not observed in control groups. Together, these findings highlight the potential of polymeric nanocarriers to modulate autoreactive immunity through Treg-mediated mechanisms [5].

Parallel to these therapeutic and immunomodulatory strategies, an expanding area of research aims to reproduce the structural and functional features of natural melanosomes. In one such approach, methylprednisolone and melanin-like polydopamine were encapsulated in ultra-deformable liposomes functionalized with the tripeptide lysine-proline-valine derived from α-MSH. Peptide-mediated targeting to MC1R increased melanin content and TYR activity in melanocytes in vitro, enhanced melanosome transfer to keratinocytes, and restored antioxidant defenses under oxidative stress. In an H₂O₂-induced vitiligo mouse model, 14 days of treatment restored macroscopic pigmentation, significantly increased catalase and superoxide dismutase activity, and normalized TNF and IL-6 levels [282]. Building on melanin biomimicry, polymeric nanoparticles composed of synthetic polydopamine have also been engineered to function as artificial melanosomes, an approach supported by the intrinsic photoprotective properties of polydopamine. In experimental models, these nanoparticles were internalized by keratinocytes and organized in a manner analogous to natural melanosomes, forming perinuclear microparasols that protected cells from UV-induced oxidative stress [283, 284].

More recently, the amphiphilic di-block copolymer poly(dimethylsiloxane)-b-poly(2-methyloxazoline) polymersomes co-loaded with TYR and L-DOPA or dopamine precursors were developed to enable controlled intravesicular polymerization of melanin or polydopamine. Confinement within the polymeric membrane prevented particle aggregation and cytotoxicity while preserving UV-absorbing properties, and both polydopamine-tyrosinase and melanin-tyrosinase formulations remained colloidally stable for several months. Notably, polydopamine-based polymersomes displayed superior UV absorption, which translated into enhanced photoprotection and increased viability of UV-irradiated cells in vitro [285].

In summary, these findings highlight nanotechnology as a promising and versatile platform for the development of future advanced therapies for vitiligo. By enhancing the targeted delivery, stability, and bioavailability of both conventional and emerging therapeutic agents, nanotechnology may enable more precise modulation of immune pathways and melanocyte regeneration, supporting the design of more effective and personalized treatment strategies for this immune-mediated disease.

Collectively, these therapeutic strategies illustrate a progressive shift from broad immunosuppression toward integrated approaches that combine immune modulation, metabolic reprogramming, and tissue regeneration.

## Final Remarks

Vitiligo is increasingly understood as a multifactorial immune-mediated disorder driven by defective immune control of autoimmune responses in the skin and impaired tissue regeneration, a framework that challenges traditional paradigms centered on broad immunosuppression. Collectively, the evidence discussed in this review supports the concept that vitiligo should be redefined not only as an autoimmune disease, but as a condition characterized by failure of immune resolution and impaired tissue regeneration, in which persistent immune memory and defective regulatory mechanisms prevent restoration of cutaneous homeostasis. Over the past decade, substantial advances in cutaneous immunology and molecular biology have expanded the therapeutic landscape to include targeted cytokine and kinase inhibition, immune checkpoint modulation, restoration of regulatory pathways, phototherapy-based immunoregeneration, pro-melanogenic agents, nucleic acid-based interventions, extracellular vesicle platforms, and cell-based regenerative strategies. Collectively, these approaches reflect a shift toward mechanism-informed interventions aimed not only at controlling inflammation but also at preserving melanocyte reservoirs and supporting functional repigmentation.

Accumulating evidence further supports combined and sequential treatment paradigms integrating immune modulation, induction of repigmentation, and long-term maintenance. However, translation of these advances into routine clinical practice remains constrained by cutaneous pharmacokinetic limitations, regulatory and manufacturing challenges, and the paucity of long-term safety and efficacy data. Indeed, a substantial proportion of these strategies remains at preclinical or early clinical stages, underscoring the need for standardized experimental models and well-designed longitudinal trials to establish long-term efficacy and safety.

The highly selective stratum corneum continues to limit the durability and effectiveness of many otherwise promising agents, particularly those requiring precise spatial and temporal control within the skin. These challenges underscore the need for standardized preclinical models, well-designed longitudinal clinical trials, and sustained interdisciplinary collaboration to advance precision-based therapies in vitiligo.

Within this rapidly evolving therapeutic landscape, nanotechnology emerges as a unifying and enabling platform rather than an isolated modality. Nanotechnology-based delivery systems offer the capacity to enhance drug stability and solubility, enable controlled and prolonged release, facilitate targeting of immune and melanocytic compartments, and improve cutaneous bioavailability while reducing systemic exposure and adverse effects. Importantly, nanocarriers provide a translational bridge for diverse therapeutic classes discussed throughout this review, including small molecules, biologics, RNA/DNA therapeutics, and regenerative agents, thus addressing key barriers that currently limit their clinical impact. While not sufficient as standalone solutions, these technologies may critically enhance the effectiveness of immunomodulatory and regenerative strategies when integrated into multimodal therapeutic approaches.

Hence, although significant translational gaps persist due to manufacturing, regulatory, and financial constraints, the convergence of nanotechnology with precision medicine and immunoengineering represents a realistic and transformative pathway toward more targeted, personalized, and effective interventions for vitiligo. Finally, future therapeutic success will likely depend on the ability to simultaneously modulate pathogenic immune memory, restore regulatory immune networks, and re-establish melanocyte regenerative niches, rather than targeting isolated disease components.

## Data Availability

No datasets were generated or analysed during the current study.
